# Sub-chronic toxicity study in rats orally exposed to nanostructured silica

**DOI:** 10.1186/1743-8977-11-8

**Published:** 2014-02-07

**Authors:** Meike van der Zande, Rob J Vandebriel, Maria J Groot, Evelien Kramer, Zahira E Herrera Rivera, Kirsten Rasmussen, Jan S Ossenkoppele, Peter Tromp, Eric R Gremmer, Ruud JB Peters, Peter J Hendriksen, Hans JP Marvin, Ron LAP Hoogenboom, Ad ACM Peijnenburg, Hans Bouwmeester

**Affiliations:** 1 RIKILT – Wageningen University & Research Centre, 6700 AE Wageningen, The Netherlands; 2 National Institute for Public Health and the Environment, 3720 BA Bilthoven, The Netherlands; 3 Joint Research Centre, 21027 Ispra (VA), Italy; 4 TNO Earth, Environmental and Life Sciences, 3508 TA, Utrecht, The Netherlands

**Keywords:** Nano, Synthetic amorphous silica, Silica, Oral exposure, In vivo, Toxicity

## Abstract

**Background:**

Synthetic Amorphous Silica (SAS) is commonly used in food and drugs. Recently, a consumer intake of silica from food was estimated at 9.4 mg/kg bw/day, of which 1.8 mg/kg bw/day was estimated to be in the nano-size range. Food products containing SAS have been shown to contain silica in the nanometer size range (*i.e.* 5 – 200 nm) up to 43% of the total silica content. Concerns have been raised about the possible adverse effects of chronic exposure to nanostructured silica.

**Methods:**

Rats were orally exposed to 100, 1000 or 2500 mg/kg bw/day of SAS, or to 100, 500 or 1000 mg/kg bw/day of NM-202 (a representative nanostructured silica for OECD testing) for 28 days, or to the highest dose of SAS or NM-202 for 84 days.

**Results:**

SAS and NM-202 were extensively characterized as pristine materials, but also in the feed matrix and gut content of the animals, and after *in vitro* digestion. The latter indicated that the intestinal content of the mid/high-dose groups had stronger gel-like properties than the low-dose groups, implying low gelation and high bioaccessibility of silica in the human intestine at realistic consumer exposure levels. Exposure to SAS or NM-202 did not result in clearly elevated tissue silica levels after 28-days of exposure. However, after 84-days of exposure to SAS, but not to NM-202, silica accumulated in the spleen. Biochemical and immunological markers in blood and isolated cells did not indicate toxicity, but histopathological analysis, showed an increased incidence of liver fibrosis after 84-days of exposure, which only reached significance in the NM-202 treated animals. This observation was accompanied by a moderate, but significant increase in the expression of fibrosis-related genes in liver samples.

**Conclusions:**

Although only few adverse effects were observed, additional studies are warranted to further evaluate the biological relevance of observed fibrosis in liver and possible accumulation of silica in the spleen in the NM-202 and SAS exposed animals respectively. In these studies, dose-effect relations should be studied at lower dosages, more representative of the current exposure of consumers, since only the highest dosages were used for the present 84-day exposure study.

## Background

Synthetic amorphous silica (SAS) is widely applied in food products within the EU as a food additive (E551) for several decades. Consumers are daily exposed to synthetic amorphous silica used in cosmetics, medicinal products, and food as an anticaking agent or carrier of flavors [1-4]. SAS is a nanostructured material composed of aggregates of primary particles in the lower nanometer size range [1,2,5-8]. Production methods of SAS include wet (precipitation) and thermal (pyrogenic) processes. SAS intended for application in foodstuffs is predominantly produced by a standard pyrogenic process in which SiCl_4_ is burned in a hydrogen flame at temperatures ranging from 1000 to 2500°C. This results in the generation of primary silica nanoparticles of ~10 nm that aggregate into particles with sizes in the order of 100 nm, which ultimately agglomerate into particles in the larger nano- or micro-size range [1,2,6].

Recently, a consumer intake of silica from food was estimated at 9.4 mg/kg bw/day, of which 1.8 mg/kg bw/day was estimated to be in the nano-size range [4]. Food products containing SAS have been shown to contain silica in the nanometer size range (*i.e.* with a size of 5 – 200 nm) in quantities up to 43% of the total silica content [4]. This fraction of SAS in the nanometer size range potentially reaches up to 100% in the small intestine based on an *in vitro* simulated digestion [9]. In this previous study, as well as in the present study, the fraction of SAS in the nanometer range has been determined with hydrodynamic chromatography inductively coupled plasma mass spectroscopy (HDC-ICP-MS), which is able to provide mass-based size distributions of SAS from food and feed matrices in the nanometer range. Sizes can be reliably determined in a size range of 5–200 nm, which is broader than the 1–100 nm size range used in most legal definitions of nanoparticles. Next to added synthetic silicon compounds, silicon also occurs as a natural component in foodstuffs in the form of sodium, calcium and magnesium silicates, or as hydrated silica SiO_2_-*n*H_2_O [10]. The latter may form small (*i.e*. 1 to 5 nm) particles that can be found in natural and drinking waters [5,11]. Reviews of toxicokinetics and toxicodynamics data of SAS generally suggest safety for consumers when exposed to SAS via food [1,2,12-14], as indicated by a LOAEL (based on liver toxicity in rats) of 1500 mg/kg bw/day [4]. However, concerns have been raised over the limited characterization of the used SAS and over the contribution (if any) of the nano-sized silica fraction to the observed effects [14]. Since the original SAS exposure studies, performed in the 1980’s, no oral *in vivo* study has been reported in the public domain. *In vivo* studies on oral exposure to SAS are required since several recent animal studies, using precipitated silica nanoparticles, show toxic effects in the liver in a particle-, size-, and dose-related manner following intravenous or intraperitoneal exposure to silica nanoparticles, which will be discussed in more detail in the results and discussion section [15-21].

The goal of the present study was to investigate the biodistribution and the effects of a pyrogenically produced food grade SAS in rats, following sub-chronic oral exposure. In addition, pyrogenic NM-202 was included in the study as a reference compound, being the OECD representative nanostructured silica for applications related to food. During the first 28 days, six groups of rats (n = 5) were daily fed a bolus of SAS or NM-202 at three different doses, namely 100, 1000, and 2500 mg/kg bw of SAS, and 100, 500, and 1000 mg/kg bw of NM-202 (Table 1; Additional file 1: Table S1). Biochemical assessment of blood from animals exposed for 28 days did not indicate systemic toxicity of the materials. Consequently, it was decided to continue the exposure to the highest dose of SAS or NM-202 for 84 days in two additional groups (n = 5). Two groups of control rats (n = 5), each corresponding to one of the exposure periods, were fed carrier material only. Of all animals, tissues and blood were collected for systemic toxicity, histological, and immunotoxicity analysis, and to assess tissue distribution. Finally, mRNA was isolated from jejunum and liver for transcriptome analysis.

**Table 1:** Intended and actual silica exposure doses

**Group**	**Intended total silica exposure dose (mg/kg bw/day)**		**Actual exposure dose**	
		**Total silica (mg/kg bw/day)**	**Added silica**^**a **^**(mg/kg/bw/day)**	**Silica in nano-size range**^**b **^**(mg/kg bw/day)**
SAS low	100	222	83	33
SAS medium	1000	942	819	328
SAS high	2500	2142	2047	819
NM-202 low	100	221	82	82
NM-202 medium	500	537	405	405
NM-202 high	1000	933	810	810
Negative control	0	133	0	<21

The actual exposure doses were calculated from the (HDC) ICP-MS silicon measurements in the feed mixture (0.95 ± 0.19 mg silica/g), standard feed pellets (1.8 ± 0.9 mg silica/g), and drinking water (0.019 ± 0.003 mg silica/g). Exposure to silica originating from consumption of standard feed pellets and drinking water was based on an average feed intake of 27 g/day and an average water intake of 45 ml/day at an average body weight of 350 g [22] (for a more detailed calculation, see Additional file 1: Table S1). ^a^Total silica (mg/kg bw/day) – (dose of silica from the standard diet + drinking water in mg/kg bw/day) = dose of added silica (*i.e.* SAS or NM-202 in mg/kg bw/day). ^b^With a size of 5 – 200 nm.

## Results and discussion

### Material characterization: pristine materials

**Figure 1:**
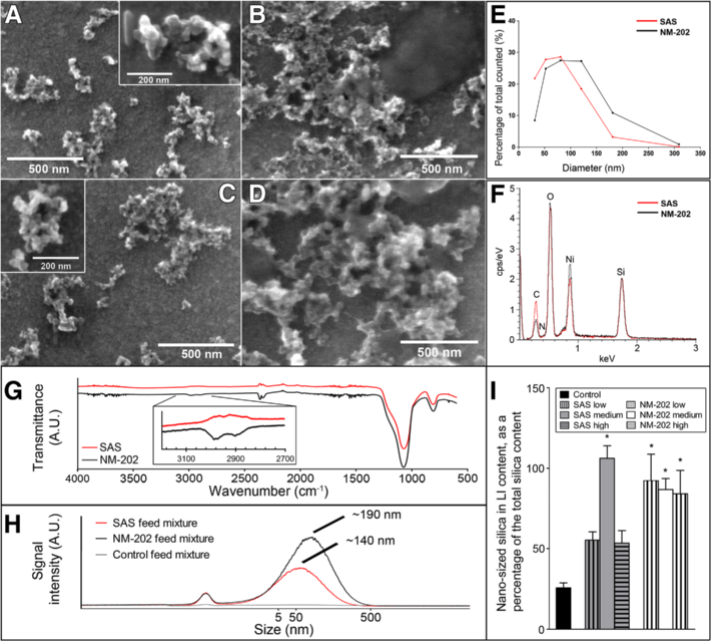
**Physicochemical characterization of synthetic amorphous silica (SAS) and the OECD representative nano-sized silica (NM-202).** SEM micrographs of SAS in **(A)** water + 0.05% BSA and **(B)** feed, and of NM-202 in **(C)** water + 0.05% BSA and **(D)** feed. **(E)** SEM size distribution pattern of SAS and NM-202 in water + 0.05% BSA, showing larger sizes for NM-202. The size limit of detection lies at 25 nm. **(F)** EDX characterization of SAS and NM-202 agglomerates in water containing 0.05% BSA, demonstrating the presence of Si, O, and C on the surface of both materials. The peak representing nickel can be attributed to the nickel coated membrane that was used for sample preparation. **(G)** FTIR spectra of SAS and NM-202 showing mild C-H stretching vibrations for NM-202 (inset). **(H)** HDC-ICP-MS chromatogram showing the size distribution and concentration (area under the curve) of nano-sized silica in SAS, NM-202, and in control feed mixtures. **(I)** The fraction of silica in the nano-size range (*i.e.* with a size of 5–200 nm; measured by HDC-ICP-MS) given as a percentage of the total silica content (measured by ICP-MS) in the large intestinal (LI) contents, 24 hours after the last exposure (mean ± standard error of the mean; n = 5). * Significant difference *versus* the control (p < 0.05).

The SAS that was used in this study is a commercially available food-grade, hydrophilic, pyrogenic synthetic amorphous silica with a primary particle size of 7 nm, a specific surface area of 380 m^2^/g, and a purity ≥99.8% (as specified by the manufacturer, see materials section). NM-202 is a representative nanostructured silica, selected by the OECD, which is also a hydrophilic, pyrogenic synthetic amorphous silica and has a primary particle size between 10 and 25 nm, a specific surface area of 200 m^2^/g, and a purity ≥99.9% (as specified by the manufacturer, see materials section). All material properties are summarized in Table 2. For exposure, SAS or NM-202 was mixed with powdered standard feed pellets and chocolate milk. Suspensions of both SAS and NM-202 in water containing 0.05% bovine serum albumine (BSA) as a stabilizing agent, showed the presence of agglomerates, which appeared to be larger in the feed mixture (Figure 1A-D), as assessed by scanning electron microscopy (SEM). SEM was also employed to generate number-based size distribution data of pristine materials, suspended in water + 0.05% BSA, which showed larger sizes for NM-202 (Figure 1E). A fraction of 78% and 61%, of the pristine SAS and NM-202 materials respectively, was below 100 nm. This could possibly be higher regarding the size limit of detection of 25 nm. The number-based size distribution of SAS and NM-202 was not assessed in the feed mixture by SEM, since the mixture would have to be extremely diluted for measurement, thereby resulting in non-representative data for this matrix.

**Table 2:** Summary of the material properties

	**Pristine material characterization**	
	**NM-202**	**SAS**
General	Hydrophilic pyrogenic^a^	Hydrophilic pyrogenic^a^
Specific surface area	200 m^2^/g^a^	380 m^2^/g^a^
Purity	≥99.9%^b^	≥99.8%^a^
Primary particle size	10-25 nm^a^	7 nm^a^
SEM size distribution	At least 61% of the material was <100 nm.^c^ (Figure 1E)	At least 78% of the material was <100 nm.^c^ (Figure 1E)
XPS	Si: 25.0 ± 0.3 at%^b^	Si: 29.8 ± 0.5 at%
	O: 72.1 ± 0.4 at% ^b^	O: 68.1 ± 0.6 at%
	C: 2.9 ± 0.6 at% ^b^	C: 2.1 ± 0.6 at%
EDX	Presence of carbon on the surface (Figure 1F).	Presence of carbon on the surface (Figure 1F).
FTIR	Large peak between 1000–1130 cm^-1^ and a peak at ~800 cm^-2^ corresponding to Si-O bonds in a pattern characteristic for amorphous fumed silica.	Large peak between 1000–1130 cm^-1^ and a peak at ~800 cm^-2^ corresponding to Si-O bonds in a pattern characteristic for amorphous fumed silica.
	Some C-H stretching vibrations at ~3000 cm^-1^, indicating the presence of organic material on the surface.	
	**Material characterisation in the feed matrix**	
(HDC)-ICP-MS	79.6 ± 20.7 mg silica/g feed, ~100% between 5–200 nm	80.5 ± 20.9 mg silica/g feed, ~40% between 5–200 nm
SEM-EDX	Presence of carbon on the material surface before and after *in vitro* digestion.	Presence of carbon on the material surface before and after *in vitro* digestion.
	Presence of salts (*e.g.* Ca and P, likely in the form of CaPO_4_) on the material surface after *in vitro* digestion_._	Presence of salts (*e.g.* Ca and P, likely in the form of CaPO_4_) on the material surface after *in vitro* digestion_._
	(Additional file 1: Figure S1)	(Additional file 1: Figure S1)
	**Material characterization in intestinal content**	
(HDC)-ICP-MS	81, 81, 97% (low, medium and high group respectively) of the material has a size between 5–200 nm	55, 106, 54% (low, medium and high group respectively) of the material has a size between 5–200 nm
Dissolution	~15-20 wt% or less dissolves after *in vitro* digestion.	~15-20 wt% or less dissolves after *in vitro* digestion.

^a^As specified by the manufacturer. ^b^Reported in ref [23]. ^c^Size limit of detection at 25 nm.

X-ray photoelectron spectroscopy (XPS) analysis of both materials identified the presence of Si, O, and C on the surface of both materials. SAS contained 29.8 ± 0.5 at% Si, 68.1 ± 0.6 at% O, and 2.1 ± 0.6 at% C, whereas NM-202 has previously been reported to contain 25.0 ± 0.3 at% Si, 72.1 ± 0.4 at% O, and 2.9 ± 0.6 at% C [23]. The presence of carbon on both materials is considered to be due to surface contamination of the particles, as also reported previously [23]. The Si : O ratio of both materials is below the theoretical value of 0.5, indicating that the surface contamination likely consists of carbon-oxygen compounds [23]. Semi-quantitative energy dispersive X-ray spectroscopy (EDX) data confirmed the XPS data, and also demonstrated the presence of carbon on the surface of SAS and NM-202 (both in water + 0.05% BSA and in the feed matrix; Figure 1F; Additional file 1: Figure S1). EDX was also used to characterize both materials in the feed matrix after *in vitro* digestion. For this, we used the previously described *in vitro* digestion procedures used to study the fate of nanoparticles during *in vitro* human digestion [9,24-26]. The *in vitro* digestion model is based on human physiological data (*i.e.* transit times, pH, and composition of digestive juices). The gastrointestinal tract is simulated for the mouth, stomach, and small intestine. The large intestine is not taken into account because *in vivo* absorption mainly takes place in the small intestine. The data indicated that, even after digestion, carbon was still present on the surface of the materials, as well as some salts (*e.g.* Ca and P, likely in the form of CaPO_4_; Additional file 1: Figure S1)_._ Fourier transform infrared spectroscopy (FTIR) analysis of SAS and NM-202 showed a large peak between 1000–1130 cm^-1^, as well as a peak at ~800 cm^-1^, which can be attributed to Si-O bonds, following a pattern that is characteristic of amorphous fumed silica (Figure 1G). The presence of isolated silanol groups on the surface of amorphous silica particles has been described to promote cytotoxicity through their ability to interact with cell membranes and to generate reactive oxygen species [27]. The spectra were therefore carefully evaluated for the presence of a peak at ~3750 cm^-1^, representative of isolated silanol groups, which was absent in both spectra. Furthermore, also a peak between 3200 – 3500 cm^-1^, corresponding to hydrogen bonded OH groups, was absent in both spectra. The small peaks in the region from 2300–2400 cm^-1^ were caused by CO_2_ interference from the air, and are therefore not of interest. Some C-H stretching vibrations at ~3000 cm^-1^ were seen for NM-202, which appeared to be the only minor difference between the two materials in the FTIR spectra. These stretching vibrations indicate the presence of organic material on the surface of NM-202, but not on the surface of SAS. Finally, C-O stretching vibrations could not be detected in the spectra, due to the presence of the Si-O peaks at the same wavenumber, which makes it difficult to directly compare the FTIR data with the XPS and EDX data with respect to the presence of carbon on the surface of the materials. The combination of these surface analyses of SAS and NM-202 revealed no, or minor differences between these two materials in neither its powdered form nor after *in vitro* digestion.

### Material characterization: SAS and NM-202 in feed matrix

HDC-ICP-MS was used to quantify the fraction of silica particles in the nano-size range (*i.e.* with a size 5–200 nm) [9] in the SAS and NM-202 feed mixtures. As a reference, total silica contents of both feed mixtures were determined by conventional ICP-MS. Since (HDC) ICP-MS measures only the Si content, all results were converted to SiO_2_, and presented as SiO_2_ throughout the text. The SAS feed mixture contained a fraction of ~40 wt% of silica in the nano-size range on a total silica content of 80.5 ± 20.9 mg/g feed mixture. The NM-202 feed mixture contained a fraction of ~100 wt% of silica in the nano-size range on a total silica content of 79.6 ± 20.7 mg/g feed mixture (Figure 1H). Lastly, the control mixture contained only 0.95 ± 0.19 mg silica/g standard feed pellet and no silica in the nano-size range. Following administration of the feed mixtures, the animals also received standard diet pellets and drinking water (*ad libitum*), containing naturally occurring silica. (HDC) ICP-MS measurements showed total silica contents of 0.019 ± 0.003 mg/g in drinking water, and 1.8 ± 0.9 mg/g in diet pellets. Silica in the nano-size range of 5–200 nm was absent. These combined data were used to calculate the actual total silica and silica in the nano-size range exposure doses (Table 1; Additional file 1: Table S1). Animals were fed standard feed pellets and drinking water containing naturally occurring silica. As stated in the introduction, the silica is likely present as soluble hydrated silica SiO_2_. *n*H_2_O and may contain small polysilicic acid particles in the size range of 1–5 nm. Due to the presence of silica in every standard diet, this approach is considered a realistic exposure. However, it must be noted that the background dose of this naturally occurring silica contributed substantially to the actual exposure dose of total but not nano-sized silica, in the low dose and control groups.

### Material characterization: SAS and NM-202 in intestinal content

One day after the last exposure, the small and large intestinal contents of the 28-day exposed animals was also analyzed by HDC-ICP-MS to get an impression of the presence of silica in the nano-size range (*i.e.* with a size of 5–200 nm) in the intestines. In the small intestine, between 50 and 100% of the total silica content was present in the nano-size range in most exposure groups *versus* 17% in the control group. However, taking the gastric and gut transition times into account, implying that most of the material has already passed the small intestine, results from the large intestinal content provide a more realistic insight into the presence of silica in the nano-size range in the gut. Therefore, only results from the large intestinal content are reported here in detail (Figure 1I; Additional file 1: Table S2). In the NM-202 exposed rats ~80% of the total amount of silica was present in the nano-size range, whereas in the SAS exposed rats this was more variable (50, 100, and 50% for the three exposure groups respectively). The control rats had the lowest fraction of 25% of silica particles in the nano-size range in the large intestinal content. The presence of silica in the nano-size range in the control rats can be explained by the observation that the control feed contains total silica (Table 1). It may be in the form of silicic acid, or in the form of large agglomerates of silica, which break up into nano-sized particles upon digestion, as observed previously [9]. These results clearly illustrate that the gut contains a substantial fraction of silica particles in the nano-size range, which appears to be 2 to 4 times higher in animals that received NM-202 or SAS.

**Figure 2:**
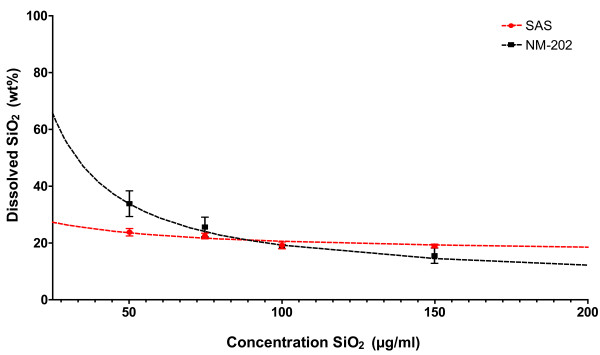
**Dissolution behavior of SAS and NM-202 after digestion *in vitro*.** The content of dissolved silica is given as a weight percentage of the total silica content at concentrations ranging from 50 to 150 μg/ml (n = 6). The dotted lines represent an extrapolated trendline for both samples. LOD: 5 μg SiO_2_/ml

Dissolution of silica has been reported before [28,29] and is described to be influenced by many parameters, including particle size, aggregation, pH [28,30], temperature, and ionic strength [30]. Therefore, the potential dissolution of SAS and NM-202 was assessed under the harsh digestive conditions, using the *in vitro* digestion approach. SAS and NM-202 suspensions in water + 0.05% BSA were digested and ultrafiltration was applied to separate the amorphous silica agglomerates from the dissolved silica. The silicon content before and after ultrafiltration was then measured by ICP-MS. Dissolution of silica results in the formation of silicic acid, which may consist of both monomeric and polymeric species [30,31]. During this process, new small particles may be formed in the solution. In natural waters, including drinking and mineral waters, these particles have been described in the size range of 1–5 nm [5]. The pore size of the ultrafiltration membrane that was used ranges between 6–12 nm [32], indicating that dissolved silica, including particles up to ~12 nm, were separated from the amorphous silica agglomerates and measured. For this experiment, the silica concentrations were ~1000 times lower than those in the original feed mixtures, which was required to prevent clogging of the ultrafiltration membrane. At these concentrations, no clogging was observed. No statistical significant differences in solubility between SAS and NM-202 were observed in the intestinal content, although it appeared that a higher percentage of NM-202 dissolved at low concentrations ≤50 μg/ml compared with SAS. However, at higher concentrations ≥100 μg/ml, dissolution of both materials appeared to stabilize at approximately 15–20 wt% (Figure 2). This indicates that the dissolved silica content in the feed mixtures in the intestine was ~15-20 wt% or possibly even slightly lower. Gastrointestinal silicic acid absorption is reported to be variable, but it is around 40 to 50%, depending on the food that contains silicic acid [33-35].

### Silica uptake in tissues

**Figure 3:**
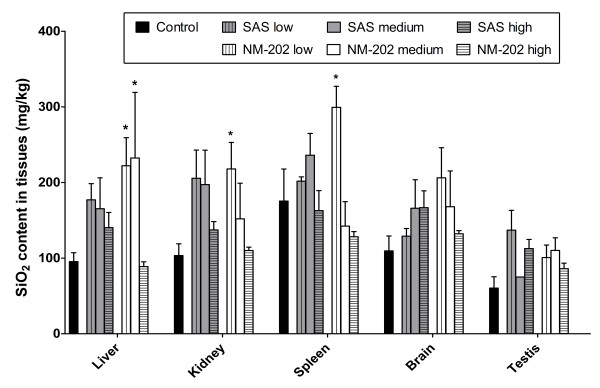
**Silica content in organs of animals orally exposed to SAS or NM-202 for 28 days.** Silica content was measured by ICP-MS and presented in mg silica/kg tissue (mean ± standard error of the mean; n = 5). * Significant difference *versus* the control (p < 0.05).

The limit of detection for silica in the nano-size range using HDC-ICP-MS was relatively high (*i.e.* 300 mg of silica in the nano-size range/kg tissue), which rendered this technique unsuitable to determine the amount of silica in the nano-size range in tissues obtained from this study. The limit of detection of SEM-EDX in a scanning mode was estimated to be lower (~100 mg silica/kg tissue) and can be even lower when examined at a single cell level. However, no nano-sized silica could be detected in the liver of exposed rats using SEM-EDX (data not shown). Therefore, only the total silica content in tissues, as determined by ICP-MS measurements, is reported here. Thus, no information in what form silica was taken up could be obtained. Intravenously or intraperitoneally administered silica nanoparticles synthesized by a precipitation process have been described to distribute mainly to the liver and spleen [18,21,36-38], but distribution to the lung [21,38], and kidney [18] have also been reported. In the present oral feeding study with SAS or NM-202, no clear target organ could be identified after 28-days of exposure (Figure 3). Only in the lower dosed NM-202 animals, significant increases in total silica concentrations were seen in the liver, kidney, and spleen. After 84-days of exposure, tissue distribution was similar to that after 28-days of exposure (Table 3), with the exception of the spleen of the animals that received the highest dose of SAS. Here, the silica content was significantly higher than that in spleens of the controls and in spleens of animals that received a high SAS dose for 28 days. Whereas clearly elevated silica levels were observed in the spleen of SAS treated animals after oral exposure to the highest dose for 84 days, exposure to the highest dose of NM-202 for 84 days did not result in accumulation of silica in any of the examined tissues. It should be noted though, that the highest dose of total silica in the NM-202 group was 2.5-fold lower that the highest dose of total silica in the SAS group. Taking into consideration that the dissolution of SAS and NM-202 could go up to 20 wt% in the intestinal content, it is possible that dissolved silica is absorbed and distributed to the examined tissues.

**Table 3:** Silica content in tissues in mg silica/kg tissue (mean ± standard error of the mean, n=5) after 84 days of exposure

	**SAS high**	**NM-202 high**	**Control**
Liver	78 ± 2	<75*	<75*
Kidney	79 ± 4	<75*	<75*
Spleen	248 ± 81^*a,b*^	<75*	<75*^b^
Brain	100 ± 23	<75*	<75*
Testis	105 ± 17	<75*	87 ± 12

^*a*^Significant increase *versus* control, or ^*b*^*versus* the results from the corresponding group after 28-days of exposure (p<0.05). *Measurements were below the limit of detection and were therefore set at the limit of detection of 75 mg silica/kg tissue.

In literature, intravenously injected silica nanoparticles (a single dose of 50, 100, 200 nm particles administered at 50 mg/kg bw [18], or of 20 and 80 nm particles administered at 10 mg/kg bw [21]), were described to be retained in liver and spleen for at least four weeks, suggesting accumulative properties of silica nanoparticles. However, direct extrapolation of these findings from studies using monodisperse silica nanoparticles to our study with nanostructured silica is difficult because of the differences in materials that were used and the differences in study design. After 28-days of exposure, total silica levels in tissues were variable, but increased up to 1.5 to 2 times in the tested tissues compared with the controls. In the liver of the NM-202 treated rats in the low and medium dose group (*i.e.* 100 and 500 mg NM-202/kg bw/day) the total silica content was significantly increased. In kidney and spleen this was observed only in rats treated with the lowest dose of NM-202.

**Figure 4:**
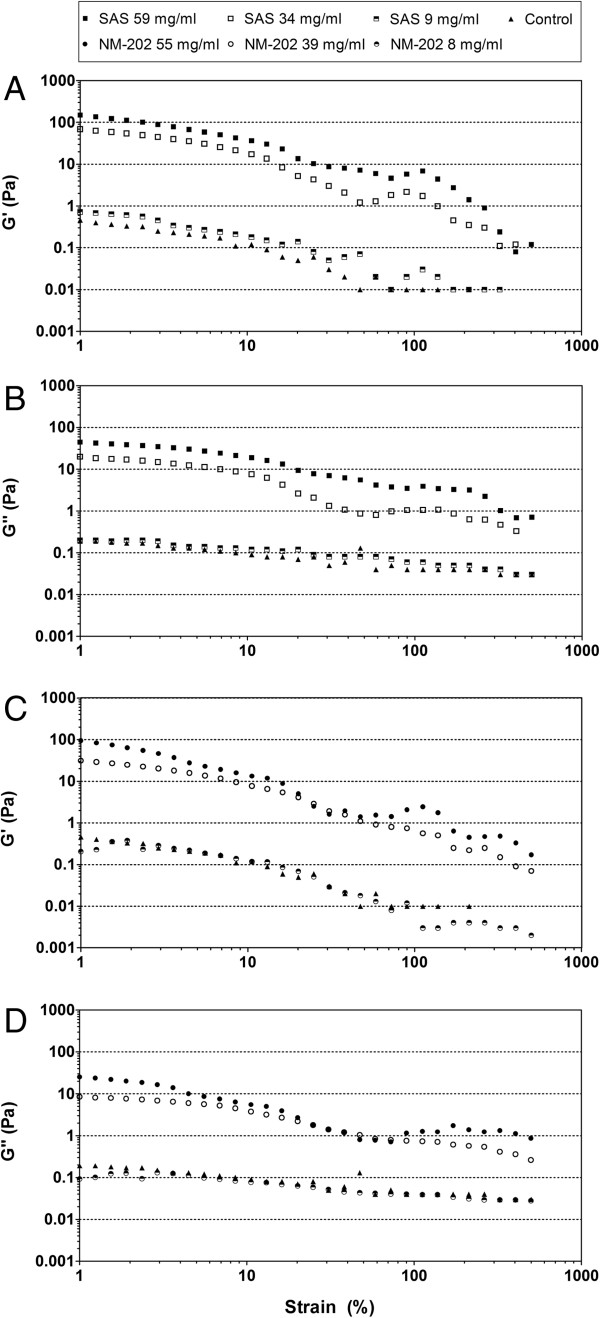
**Visco-elastic behavior of SAS and NM-202 in the feed mixture after *in vitro *digestion.** Higher storage moduli (G’) of **(A)** SAS and **(C)** NM-202 than loss moduli (G”) of **(B)** SAS and **(D)** NM-202 (as a function of the strain) indicate increasing gel-like properties, with increasing SiO_2_ concentrations.

While not statistically significant, the total silica content in tissues appeared to be lower in the higher-dosed animals compared with the lower-dosed animals, in particular for NM-202. This can be explained by the gelating behavior of silica, which has been described to occur more readily at higher particle concentrations under conditions with a relatively high pH and salt concentration, like in the small intestine [39]. In order to examine this in more detail, the visco-elastic behavior of SAS and NM-202 was analyzed by rheological measurements at different concentrations after digestion *in vitro*. Since silica measurements in the intestinal contents were performed 24 hours after the last exposure, these data likely underestimate the maximal silica concentration. Therefore, silica concentrations in the intestines were estimated based on physiological data from literature (*i.e.* approximate secretion of mouth, gastric, and intestinal fluids; http://www.interspeciesinfo.com/), indicating that concentrations of 10, 50 and 75 mg/ml after *in vitro* digestion corresponded to the low, medium, and highest intestinal exposure doses of SAS. However, the actual silica concentrations after *in vitro* digestion were somewhat lower (*i.e.* 9, 34, and 59 mg/ml for SAS and 8, 39, and 55 mg/ml for NM-202) due to necessary pH adjustments during the *in vitro* digestion procedure. The higher storage moduli (G’) in comparison with the loss moduli (G”) for SAS and NM-202 indicate that all samples have gel-like properties (Figure 4). The control feed mixture also showed mild gel-like properties, which is probably due to the presence of chocolate milk in the mixture. The G’ and G” of the control sample were slightly lower than, or equal to the lowest SAS and NM-202 doses. Increasing silica concentrations however, led to an increased G’ and G”, showing stronger gel-like properties for the higher silica concentrations. Previously, a lower absorption of nanoparticles from gels has been described [40], suggesting that the absorption of silica might have been higher in the low and mid dose groups as compared with the high dosed groups. Considering the estimated human exposure to SAS (*i.e.* 9.4 mg/kg bw/day) and silica in the nano-size range (*i.e.*1.8 mg/kg bw/day) [4], which are both lower than the concentrations used for the rheological measurements, a low gelation of silica in the human intestine might be expected.

### Assessment of systemic and immunotoxic effects

Daily monitoring of body weights and tissue weights after dissection (Additional file 1: Table S3-4) did not indicate treatment related effects, or any effects indicative of nutritional imbalance in the animals that had received relatively high amounts of the food mixture (*i.e.* the high dose groups). Since systemically administered silica nanoparticles, synthesized by a precipitation process, have been described to distribute to the liver and kidney [18,21,36-38], also blood biochemical markers of hepatic and kidney injury were examined. Markers evaluated for hepatic injury were alkaline phosphatase (ALP), alanine transaminase (ALT), aspartate transaminase (AST), and total protein. The markers creatinine and urea were used to evaluate kidney function. After 84-days of exposure, the levels of lactate dehydrogenase (LDH), uric acid, zinc, iron, high- and low-density lipoprotein, cholesterol, glucose, and triglycerides were measured additionally to evaluate general tissue damage and to further evaluate liver damage. Neither after 28-, nor 84-days of exposure were there any signs of systemic toxicity (Additional file 1: Figure S2 A-O). ALP levels after 28-days of exposure were slightly increased in the low and high (but not medium) NM-202 dose groups compared to the control group, but remained within normal physiological ranges. Moreover, the observed decrease in LDH concentration in the NM-202 84-day exposure group did also not indicate toxicity, since only increased LDH levels are associated with toxicity. These results are in contrast with previous reports on effect after intravenous or intraperitoneal administration of silica nanoparticles synthesized by a precipitation process, which showed a dose-dependent increase in ALT and AST levels for monodisperse 70 nm silica nanoparticles (starting at 20 mg/kg bw) and increased ALT levels for 110 nm silica nanoparticles (at 50 mg/kg bw) [16,17,19]. However, the tissue silica concentrations in these studies are expected to be much higher than in the present study.

Immunotoxic effects due to any of the treatments were also absent after both 28-, and 84-days of exposure. No effects were seen on antibody levels (IgG and IgM) in blood (Additional file 1: Figure S2 P, Q), or on cytokine levels produced by proliferating T- and B-cells, that were isolated from spleen and MLN in the 28-, and 84-days exposure groups (Additional file 1: Table S5-8). Proliferation of the isolated T- and B-cells, and the activity of NK-cells isolated from spleen was also examined after 28-days of exposure, but remained unaffected (Additional file 1: Figure S2 R-V).

### Histological and transcriptome analysis

Histopathological evaluations were performed on jejunum, liver, kidney and spleen. In addition, whole genome differential mRNA expression was analyzed in jejunum epithelial tissue and liver tissue of all animals. Histopathological assessment of the kidneys and spleen showed no differences between the treated animals and the controls (data not shown). In jejunum, a quantitative measurement of the villus height and crypt depth demonstrated a small but significant increase in villus heights and crypt depths, but no significant differences in the ratio between the villus height and crypt depth for both SAS and NM-202 treated animals after 28-days of exposure, as compared with the controls (Additional file 1: Figure S3). Most absorption takes place in the jejunum, and generally speaking, long villi and a high villus:crypt ratio indicate a highly differentiated and active tissue. Gene set enrichment analysis on microarray data of jejunal epithelial samples from either the 28-, or 84-days of exposure to both SAS and NM-202 did not show differences in gene expression profiles between the treatment groups and the controls (data not shown).

**Figure 5:**
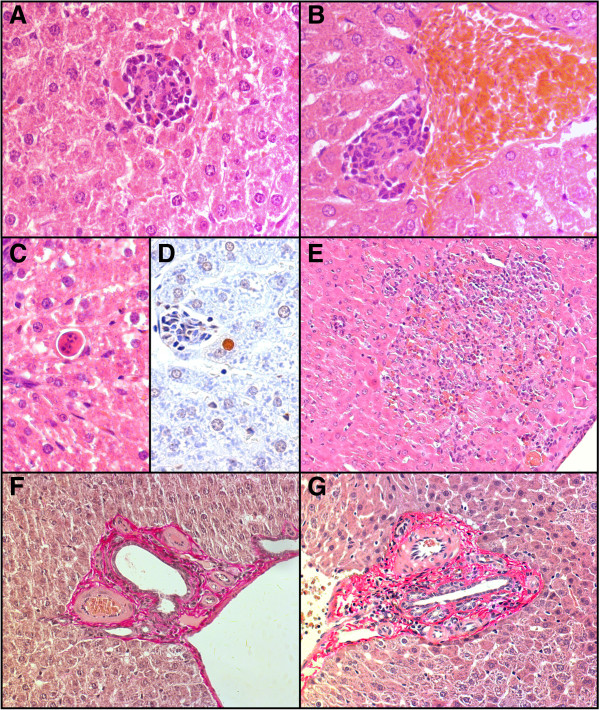
**Histological images of livers from animals treated with SAS or NM-202 for 28 or 84 days. (A, B)** Light microscopic images of an inflammatory granuloma after 84-days of exposure for **(A)** SAS high dose (magnification: 200x), and **(B)** NM-202 high dose (magnification: 200x). **(C)** Apoptosis after 28-days of exposure (SAS low dose, H&E staining; magnification: 200x), and **(D)** apoptosis after 28-days of exposure (NM-202 high dose; immunohistochemically stained apoptosis; magnification: 200x). **(E)** Necrosis after 28-days of exposure (NM-202 medium dose; magnification: 25x), and **(F, G)** fibrosis after 84-days of exposure to the **(F)** SAS high dose (magnification 100x), and **(G)** NM-202 high dose (magnification 100x).

**Figure 6:**
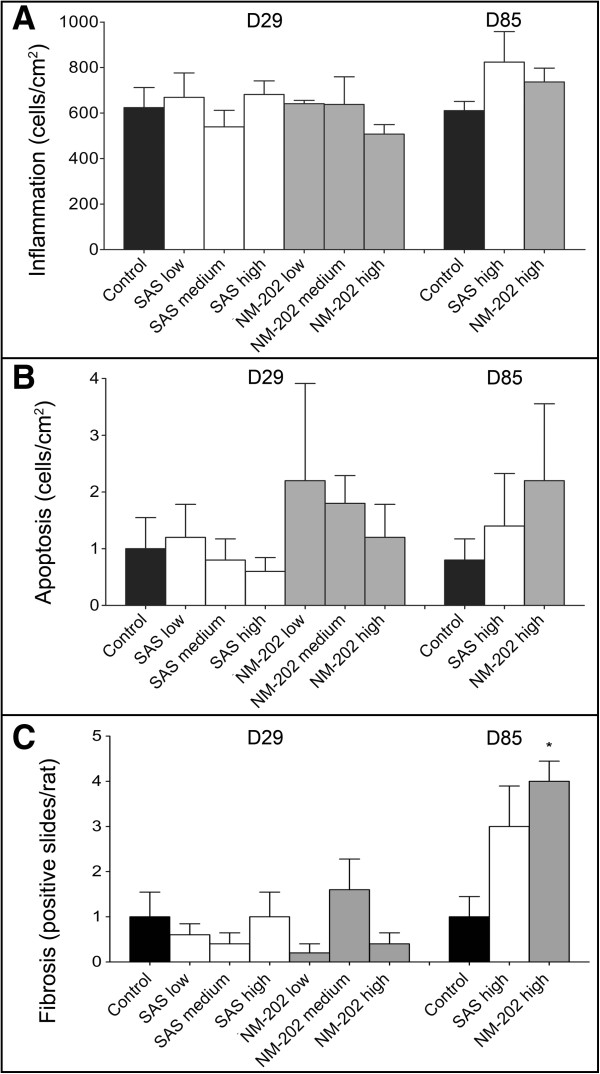
**Histopathological evaluation of livers from animals treated with SAS or NM-202 for 28 or 84 days. (A)** The number of mononuclear inflammatory cells (given per cm^2^) in liver tissue after 28-, or 84-days of exposure (mean ± standard error of the mean; n = 5). **(B)** The number of apoptotic cells (given per cm^2^) in liver tissue after 28-, or 84-days of exposure (mean ± standard error of the mean; n = 5). **(C)** The number of slides (out of a maximum of 10 evaluated slides) in which fibrosis occurred. * Significant difference *versus* the control at that day.

A quantitative histological assessment of livers indicated that the number of lymphocytic cells (Figure 5A and B) and thereby also the number of inflammatory granulomatous foci (the average number of cells in each of the foci was constant at ~19 cells/focus) remained unchanged after 28, and 84-days of exposure (Figure 6A). Furthermore, also the number of apoptotic cells (Figure 5C and D) in the livers was not significantly affected by the 28-, or 84-day treatment (Figure 6B). Necrosis (Figure 5E) was only occasionally seen and there were no differences between groups (data not shown). Contradictory, previous reports described inflammation [15,17-21], lymphocytic infiltration [17,20], increased apoptosis [17,20], necrosis [16,19-21], and silicotic nodular-like lesions [17] in the liver as a result of intravenous or intraperitoneal administration of monodisperse silica nanoparticles produced by a precipitation process of 15 nm (50 mg/kg bw) [20], 20 and 80 nm (10 mg/kg bw) [21], 30 (10 mg/kg bw), and 70 nm (40 mg/kg bw) [19], 70 nm (10, 30 mg/kg bw) [15,16], 110 nm (25, 50 mg/kg bw) [17], or 100 and 200 nm (50 mg/kg bw) [18]. This difference, between our observations and literature, is most likely caused by the use of different administration routes, potentially leading to much higher internal exposures. It could also be due to the use of nanoparticles that were synthesized by a precipitation process, possessing different physicochemical properties.

**Figure 7:**
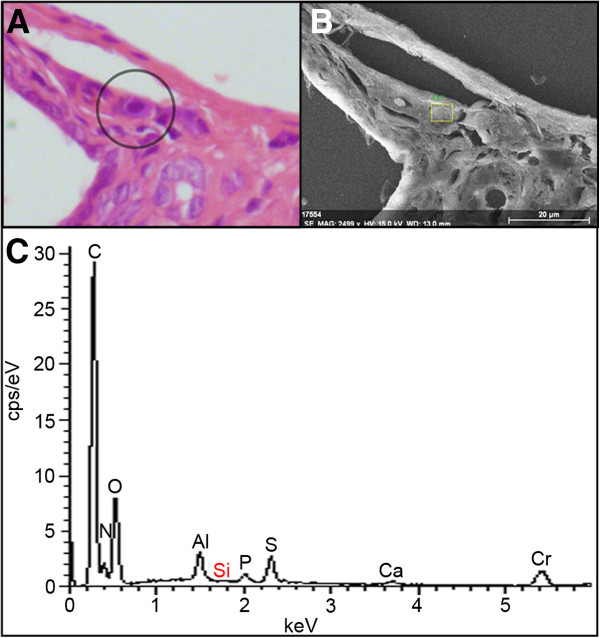
**Histological and electron microscopical images from the liver. (A)** Light microscopic image of a macrophage (indicated by the circle) in liver tissue from an animal treated with the highest dose of SAS for 84 days, and **(B)** a corresponding SEM-EDX graph of the same macrophage (indicated by the rectangle) in which **(C)** the elemental composition was analyzed.

After 84-days of exposure, the occurrence of periportal fibrosis in the liver (Figure 5F and G) was significantly increased in the NM-202 treated animals (p = 0.021), as compared with the control animals (Figure 6C; Additional file 1: Table S9). In the SAS treated animals the presence of fibrosis appeared to be increased too, but this was not significant (p = 0.073). In literature, liver fibrosis was reported in animals receiving monodisperse silica nanoparticles, synthesized by precipitation, with a size of 70 nm intravenously, at the lowest repeated dose of 10 mg/kg bw every 3 days for 4 weeks [16]. Furthermore, previous studies have suggested specific uptake of silica particles, produced by precipitation, in liver macrophages [16,36,41]. However, we could not confirm this by using SEM-EDX analysis on liver slides (Figure 7).

**Figure 8:**
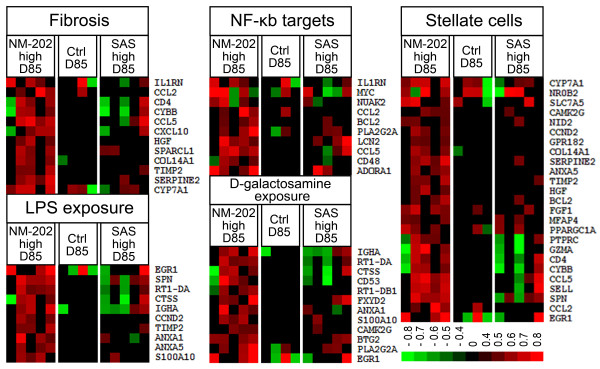
**Transcriptomic analysis of livers from animals treated with SAS or NM-202 for 84 days.** Heatmaps represent gene expression profiles of gene sets related to fibrosis in liver tissue samples after 84-days of exposure. The red and green colours indicate up- or down-regulation of gene expression (2 log expression ratio) for each individual rat in the treatment groups (n = 5) and in the control group (n = 4) *versus* the average expression of that gene in the control group. Only genes that were up- or downregulated > |1.2x| *versus* the average control in ≥3 out of the 5 rats were selected. The results indicate up-regulated fibrosis related gene expression in the NM-202 treated animals after 84 days of exposure. Comparison of gene expression in the individual control rats *versus* the average gene expression of all control rats, indicated that there was a low variation in gene expression within the control group.

Whole genome gene expression analysis was performed on mRNA isolated from liver homogenates of samples from animals exposed to SAS and NM-202 for 28-, or 84-days. In concordance with the histopathological data, gene set enrichment analysis did not reveal a significant upregulation of gene expression in gene sets correlated to inflammatory processes (data not shown). Also expression analysis of individually selected genes coding for cytokines involved in inflammatory processes that were previously shown to be affected by exposure to silica nanoparticles [16,17,20,21,42] did not show significantly altered gene expression levels (Additional file 1: Table S10). Further analysis revealed a significantly induced gene expression in a fibrosis-related gene set for samples of NM-202 treated animals after 84-days of exposure, but not for SAS treated animals (Figure 8). Comparison of gene expression in the individual control rats *versus* the average gene expression of all control rats, indicated that there was low variation in gene expression within the control group. It should be noted that, although the observed induction of gene expression in these gene sets was significant in the NM-202 treated animals, the observed differences in gene expression at a single gene level were low (*i.e.* as demonstrated in Figure 8, a threshold was set at > |1.2x| with a maximum increased expression of 5.4x *versus* the average control) and no effects were observed in any of the treatment groups after 28-days of exposure. Nevertheless, gene set enrichment analysis also showed significant enrichment of gene sets related to activated hepatic stellate cells, NF-κB target signaling, and D-galactosamine *(i.e*. a single *i.p.* exposure dose of 3000 mg/kg for 24h) or LPS (*i.e.* a single *i.p*. exposure dose of 3 mg/kg for 24h) treatment in the NM-202 treated animals after 84-days of exposure (Figure 8). These gene sets can be directly connected to liver fibrosis. Activated hepatic stellate cells are involved in the production of extracellular matrix proteins like collagen, leading to the formation of fibrotic tissue, while NF-κB plays several important roles in the development of liver fibrosis [43]. Treatment with D-galactosamine and LPS has been described to induce liver fibrosis in rodents [43]. Yet, biochemical blood parameters related to liver intoxication were not affected. This might be indicative of only generally mild effects. Alternatively, it has been shown in animal models, in which chronic fibrosis of the liver was induced by intraperitoneal injection of dimethylnitrosamine, that chronic liver fibrosis can be accompanied by base levels of AST in blood [44].

In summary, quantitative histopathological analysis showed no significant biological effects in the SAS treated animals. In the NM-202 treated animals, an induction of fibrosis in the liver was observed after 84 days of treatment, while no silica accumulation was detected in the liver. This observation was in line with the outcome of transcriptome analysis, showing an induced gene expression in fibrosis-related gene sets in the NM-202 treated animals after 84 days. At the single gene expression level this induction in gene expression remained relatively low. Taken together, these results point toward the induction of biological effects in the liver by NM-202 treatment, but the biological relevance of the observed responses requires further study.

While the SAS and NM-202 powdered materials used in this study are both produced pyrogenically, both materials differ in some aspects. SAS and NM-202 have a different specific surface area (*i.e.* 380 and 200 m^2^/g for SAS and NM-202 respectively). The silica fraction in the nano-size range in the feed mixtures used in this study was larger in the case of feed mixtures containing NM-202 than SAS, as determined by HDC-ICP-MS, and the carbon content on the surface of the materials might have been slightly different between the two materials, as determined by XPS, EDX and FTIR. After 28 days of exposure, none of the rats showed clearly elevated levels of total silica content in tissues, except for the lower dosed NM-202 animals that showed significant increases in total silica concentrations in liver, kidney, and spleen *versus* the controls. Accumulation of silica in tissues after 84 days of exposure was demonstrated for the SAS exposed animals, but not for the NM-202 exposed animals. The presence of nano-sized silica in liver could not be detected with SEM-EDX analysis in any of the treated rats. Finally, no systemic toxicity or immunotoxicity was observed.

## Conclusions

We conclude that, oral exposure of rats to NM-202 resulted in biological effects on the liver after 84 days of exposure, whereas exposure to SAS did not, which might have been caused by minor variations in the starting material (*i.e.* surface area, carbon content on the surface, and the amount of silica in the nano-size range in the feed matrix). However, at present it is not clear how these different material properties (see Table 2) relate to the observed effects. While not statistically significant, the total silica content in tissues appeared to be lower in the higher-dosed animals compared with the lower-dosed animals, in particular for NM-202. Due to technical limitations of detection equipment we could not determine the amount of silica in the nano-size range in tissues from the exposed animals, nor elucidate in which form silica was taken up. Clearly, the liver effects that were observed in the present oral exposure study are much lower in severity and incidence than in previous studies reported by others, in which silica nanoparticles (produced by precipitation) had been systemically administered. The observed liver effects appeared to be mild and were not accompanied by changes in biochemical markers in blood, but supported by mild changes in transcriptome analysis data from the liver. Additional studies seem warranted to further evaluate the biological relevance of the observed fibrosis in liver of NM-202 exposed animals and possible accumulation of silica in the spleen of SAS exposed animals. Using *in vitro* digestion studies, we showed that the intestinal content of the mid and high dosed groups had stronger gel-like properties than the intestinal content of the lowest dose groups. This implies also low gelation of silica in the human intestine, and high bioaccessibility of the silica at realistic consumer exposure levels. Therefore, future studies should include lower dosages more representative of current human exposure, since only the high doses of SAS and NM-202 were used for the 84-day exposure in the present study.

## Methods

### Study materials and preparation of animal feed

A commercially available food additive, hydrophilic pyrogenic synthetic amorphous silica (SAS) with a primary particle size of 7 nm, a specific surface area of 380 m^2^/g, and a purity of 99.8% was used, kindly donated by Evonik Degussa GmbH (Frankfurt, Germany). In addition, Joint Research Centre (JRC, Ispra, Italy) Nanomaterials Repository; hydrophilic pyrogenic silica (NM-202) was used, which was kindly donated by the JRC of the European Commission. NM-202 has a specific surface area of 200 m^2^/g, a purity of 99.9%, and a primary particle size between 10 and 25 nm.

SAS or NM-202 was mixed with standard feed and chocolate milk was added to increase palatability, which was assessed in a pilot experiment. SAS or NM-202 was mixed by hand-stirring to a thick paste with chocolate milk (Chocomel, Nutricia, The Netherlands) and ground standard diet pellets (RMH-B, ABDiets, The Netherlands) in a ratio of 1:8:1 respectively, by weight. Total intended silica content of the mixtures (both SAS and NM-202) was 99 mg/g and was fed in different amounts to rats to achieve the desired daily dosage (Table 1; Additional file 1: Table S1). Higher dosed animals were offered more of the feed mixture than lower dosed animals. All animals of each group daily consumed the complete amount of food mixture that was offered within the two hour exposure time frame. For control groups, feed without SAS or NM-202 was prepared, containing only chocolate milk and ground standard diet pellets. To adequately compensate for the amount of chocolate milk, an average amount of chocolate milk as offered to the treated animals was chosen. This resulted in a ratio of 3:2 of chocolate milk and ground pellets respectively, by weight. Thus, animals in the different treatment groups received the following amounts of chocolate milk through feeding of the feed mixtures;*.* 0.8, 8.1 and 20 g/kg/ bw/day for the SAS low, medium, and high dose groups respectively, 0.8, 4.1 and 8.1 g/kg bw/day for the NM-202 low, medium, and high dose groups respectively, and 6.1 g/kg bw/day for the control animals. The feed mixtures were prepared freshly three times a week.

### *In vivo* experimental design

Six-week-old male specific pathogen free Sprague–Dawley rats were purchased from Harlan (Horst, The Netherlands). Animals were individually housed in polycarbonate cages with cage enrichment and were allowed to acclimatize for three weeks before the start of the experiment. Room temperature was ~20°C with a relative humidity of ~55%. Individual housing was necessary, because rats were individually offered prepared food with SAS, NM-202 or vehicle. A reversed 12-h light/dark cycle was used to feed the rats in their active period and feed and water was given *ad libitum*, except for a two hour fasting period before the prepared feed was offered to the animals, and the following exposure period. Rats were allowed to consume all prepared feed, immediately thereafter the animals were being offered standard feed pellets again. During the entire study, rats ate all silica containing food mixtures or vehicle mixtures during the exposure period of two hours. The study was performed according to the national guidelines for the care and use of laboratory animals after approval of the animal welfare committee of Wageningen University.

At the start of the experiment the average body weight of the 9 weeks old animals was ~280 g and rats were randomly divided into 10 groups (n = 5). Seven groups of rats were fed SAS or NM-202 in different dosages or vehicle for 28 days, in addition the highest dosed groups of SAS and NM-202 and a control group were fed for 84 days. The groups for 28-day exposure were: 1) SAS; 100 mg/kg bw/day, 2) SAS; 1000 mg/kg bw/day, 3) SAS; 2500 mg/kg bw/day, 4) NM-202; 100 mg/kg bw/day; 5) NM-202; 500 mg/kg bw/day; 6) NM-202; 1000 mg/kg bw/day and 7) control. For the 84-day exposure, the groups were divided into: 8) SAS; 2500 mg/kg bw/day, 9) NM-202; 1000 mg/kg/day and 10) control. Dosages were chosen around the previously observed LOAEL of 1500 mg/kg bw/day for SAS [4]. The medium and high doses of NM-202 were chosen to be lower than those of SAS, because the material characterization showed a higher fraction of silica in the nano-size range in the feed matrix for NM-202. All rats were weighed daily. One day after the last exposure of the 28-day exposure groups (*i.e.* the first 7 groups), the animals were euthanized by CO_2_/O_2_ inhalation and the following organs were excised aseptically, weighed and placed on ice: liver, kidneys, spleen, brain, testis and the MLNs. Parts of the liver and the epithelium of the jejunum were also stored in liquid nitrogen, and parts of the jejunum, liver, kidney and spleen were fixed in 10% formalin. Furthermore, blood was collected on heparin and stored on ice, as well as the stomach, small (duodenum, jejunum, ileum) and large intestinal contents. One day after the last exposure of the 84-day exposure groups (*i.e.* group 8–10), all animals were euthanized and organs collected according to the same protocol as applied for the 28-day exposure groups.

Total silicon content was determined with inductively coupled plasma mass spectroscopy (ICP-MS) in liver, kidney, spleen, brain and testis. Furthermore, hydrodynamic chromatography (HDC) ICP-MS was applied to detect silica particles in the nano-size range (*i.e.* with a size of 5 – 200 nm) in gastrointestinal contents. Systemic toxicity was monitored by analysis of biochemical markers in serum and by histopathological analysis of jejunum, liver, kidney, and spleen. Immunotoxicity was evaluated by measuring antibody levels in blood, analysis of the proliferation of T- and B-cells isolated from the spleen end mesenteric lymph nodes (MLN) in response to lipopolysaccharide (LPS) or concanavalin A (Con A), by evaluating cytokine levels in culture media from these proliferating T- and B-cells, and by measuring the activity of natural killer (NK)-cells isolated from the spleen.

### Material characterization

Both SAS and NM-202 were characterized in aqueous suspensions by SEM. SAS and NM-202 were suspended in LC/MS grade water (Biosolve, Valkenswaard, The Netherlands), containing 0.05% BSA as a stabilizing agent, to a concentration of 10 mg/ml. Suspensions were vortexed for 1 min at full speed, followed by sonication at 20°C at 100% output (4 W specific ultrasound energy (240 J/m3), using a Branson 5510 water bath sonicator (Emerson, USA) for 30 min. Next, the suspensions were further diluted to a final concentration of 10 μg/ml in LC/MS grade water (Biosolve) and sonicated again at 20°C at 100% output in a water bath sonicator (Emerson) for 30 min. Furthermore, SAS and NM-202 were characterized in the feed matrix prepared as described earlier and diluted 100 times. For SEM measurements, droplets of the suspensions were put on a nickel coated Nuclepore track-etched polycarbonate membranes and analyzed with high-resolution field emission gun scanning electron microscopy (FEG-SEM) on a Tescan MIRA LMH FEG-SEM operated at 15 kV in combination with a Bruker EDX spectrometer with a XFlash 4010 detector with an active area of 10 mm^2^ and super light element window (SLEW), which allows X-ray detection of elements higher than borium (Z > 5). The spectral resolution of the detector is 123 eV (Mn (10kcps) ave FWHM). The SEM was equipped with a Scandium SIS software package (Olympus Soft Imaging Solutions GmbH, Germany) for automated particle analysis. With this system the filter area is automatically inspected on a field-by-field basis. In each field of view particles are recognized using a pre-selected grayscale video threshold (detection threshold level) to discriminate between a particle and the filter background. The analyses were conducted using the secondary electron (SE) mode. The particle size distribution is based on the projected area equivalent diameter (dpa). Magnifications of 25.000X (image area: 6 × 8 μm) and 75.000X (image area: 2 × 2.7 μm) were chosen in order to cover the full size range from 25–400 nm. Per size bin (25–40, 40–65, 65–100, 100–160, 160–250, 250–400 nm) a minimum of 50 particles was measured; in total more than 1000 particles were measured. EDX analysis of the material surface included a correction for the background signal. This was performed by subtraction of the background signal, using nickel as a reference element (which is present as a thin layer on the filter).

IR spectra of both materials were acquired on a Bruker Tensor 27 FTIR spectrometer equipped with a single reflection Platinum ATR accessory, at a resolution of 2 cm^-1^.

XPS characterization of NM-202 was reported previously [23]. SAS characterization was performed at the same institute, using the same protocol and equipment. The measurements (consisting of four technical replicates) were performed using an AXIS ULTRA Spectrometer (KRATOS Analytical, UK) and Vision2 software (Kratos Analytical, UK) was used for data processing. The XPS analysis provides information on the surface composition of the analyzed material (down to a depth of 10 nm) with a detection limit of ~0.1% of the atoms and an estimated 10% accuracy in the measurement of elemental compositions. More detailed information regarding the measurement is given in Additional file 1.

### *In vitro* digestion of feed samples

The used *in vitro* digestion model has previously been described [9,24-26]. Briefly, the model consists of three phases; the saliva, gastric, and intestinal phase. The dissolution and gelating behavior of SAS and NM-202 in the intestinal environment was studied after the materials passed all three phases. All artificial juices for the digestion experiments were prepared on the day before the actual digestions. The pH values of the juices were checked and, if necessary, adjusted to the appropriate interval with NaOH (1M) or HCl (37% w/w). The constituents and concentrations of the various synthetic juices are as shown in Additional file 1: Table S11. Before the start of the digestions, all digestive juices were heated to 37 ± 2°C and incubations are carried out in a head-over-head rotator at 37 ± 2°C. Experiments for the rheological measurements were performed in duplo, and six replicate samples were used for the dissolution behavior experiments.

For the rheological measurements the digestion started by introducing 2 mL of artificial saliva to the SAS/NM-202/control feed mixture. The feed mixture consisted of 8 g of chocolate milk, 1.0 g of powdered standard diet, and 0–1.0 g of SiO_2_, dependent on the desired final concentration in the intestinal phase (*i.e.* 0, 10, 50, 75 mg/mL). This mixture was rotated head-over-head for 5 min at 55 rpm at 37 ± 2°C. Subsequently, 4 mL of gastric juice was added, the pH adjusted to pH 2.0 ± 0.5, and the mixture was rotated head-over-head for 2 h at 37 ± 2°C. Finally, 4 mL of duodenal juice, 6 mL of bile juice, and 0.7 mL of NaHCO_3_ solution were added. The pH was adjusted to pH 6.5 ± 0.5, and the mixture was again rotated head-over-head for 2 h at 37 ± 2°C. Subsequently, the samples were used for rheological measurements. Additionally, a subsample of the suspensions was taken for SEM-EDX analysis to characterize the material after *in vitro* digestion.

For the dissolution measurements, SAS and NM-202 suspensions of 1 mg SiO_2_/mL were prepared in Milli-Q with 0.05% BSA. Subsequently, 630–2115 μL of these dilutions, depending on the desired final concentration in the intestinal phase (*i.e.* 50, 75, 150 μg/mL) were added to the first phase of the digestion model (i.e. 2 mL of artificial saliva). The digestion procedure was performed as described above. At the end of the digestion experiment, 4 mL of all samples were ultrafiltered by centrifugation through a cellulose filter with a nominal cutoff value of 3 kDa (Ultra-4, Amicon). The total silica content in the unfiltered and filtered suspensions were measured by ICP-MS.

### Rheological measurements

The visco-elastic behavior of the SAS, NM-202, and control feed mixtures following *in vitro* digestion was analyzed by rheological measurements. Oscillatory measurements were performed using a Physica MCR 301 (Anton Paar, Austria) stress controlled rheometer with a concentric double gap cylinder geometry (DG 26.7) to determine the storage modulus (G’) and loss modulus (G”) of the samples. The samples were subjected to a strain sweep with strains ranging from 1-500%, at a frequency of 1 Hz.

### Determination of silica (in the nano-size range) by (HDC) ICP-MS

The size and concentration of silica in the nano-size range (*i.e.* between 5–200 nm) was determined by HDC-ICP-MS in the feed mixtures and in the stomach, small and large intestinal contents of the rats after 28 days of exposure. Samples were homogenized by mixing and a subsample was collected for analysis. The subsample was sonicated in LC/MS grade water to prepare an aqueous suspension, which was filtered through a 5 μm filter (Acrodisc, Pall Lifer Sciences, USA) before HDC-ICP-MS analysis.

The HDC system was a Thermo Scientific Spectra system P-4000 liquid chromatograph (Waltham, MA, USA) equipped with a PL-PSDA HDC cartridge, type 1, length 800 mm, diameter 7.5 mm, packed with non-coated, non-porous silica spheres (Agilent Technologies, Wokingham, UK). The eluent was an aqueous 10 mM solution of sodium n-dodecyl sulphate (SDS) with a flow rate of 1.0 ml/min. Sample injection volumes were 50 μL. The ICP-MS was a Thermo X Series 2 (Waltham, MA, USA), equipped with an autosampler, a Babington nebulizer and operated at an RF power of 1400 W. Data acquisition was performed in the selected ion monitoring mode monitoring m/z ratios of 28 and 29 that are characteristic for silicon. Polyatomic interference at these ion masses is unavoidable and resulted in a high, but relatively stable, background signal due to N_2_. Acquiring data in the helium collision mode did not improve the signal/noise ratio and was not applied since it resulted in an overall lower sensitivity. The Si signal of peaks in the chromatograms was isolated by subtracting the background signal of the baseline in the same chromatogram. Aqueous suspensions of silica nanoparticles with sizes ranging from 32 to 500 nm (Microsil microspheres, Bangs Laboratories, USA) were used to calibrate the size separation of the HDC column, while a standard of 32 nm silica nanoparticles was used for quantitation and checking system performance. Data are presented as a weight percentage of silica in the nano-size range relative to the total amount of silica in the same samples.

The total silicon content was determined in the standard rat feed pellets, the prepared feed, with or without SAS or NM-202, in the drinking water, in the stomach and gut content and in the tissues. For this, 1–2 grams of sample was added to a perfluoroalkoxy digestion vial, followed by the addition of 6 ml 70% nitric acid and 1 ml of 40% hydrogen fluoride. The samples were digested in a microwave system for 45 min at ~250°C, 70 bar. Following digestion and cooling to room temperature, MilliQ water was added to a total volume of 150 ml. This solution was shortly shaken by hand and two times further diluted to a total volume of 300 ml. Finally, the extracts were analyzed by ICP-MS using the same system and settings as described above. The use of glass equipment during sample preparation and (HDC) ICP-MS analysis was avoided to prevent Si contamination. Also internal control samples were evaluated to assure the absence of Si contamination. Si measurement data were converted to SiO_2_ and presented as SiO_2_ throughout the text. The limit of detection in tissues, the stomach, small and large intestinal contents was set at 35 mg Si/kg (75 mg SiO_2_/kg), with a measurement error of ± 15 mg Si/kg for concentrations between 35 and 100 mg Si/kg and 20% at concentrations >100 mg/kg. In water and intestinal juices (for the *in vitro* digestion experiments) the limit of detection was set at 5 μg/ml based measurements in blanks.

The performance of the HDC-ICP-MS analysis in terms of response and retention time stability was determined as described earlier [9] as the standard deviation in the average response and retention time of the quantitation standard in each analysis series. The reproducibility standard deviation of the response of the quantitation standard was 20%, and that of the retention time window <2%. Compared to usual contaminant analysis the reproducibility standard deviation of the response is relatively high for two reasons. First, the ICP-MS signal for Si suffers from a high background due to the presence of N_2_ and CO, which has to be subtracted to isolate the true Si signal of the analytes. Secondly, since a “size range” has to be determined no clear narrow peaks as in regular gas or liquid chromatography are observed, but broader peaks depending on the size distribution of the particles.

The recovery of nano-sized silica (by HDC-ICP-MS) is determined by spiking blank samples with nano-sized silica and analyzing these control samples with the actual samples. The average recovery of the added nano-sized silica (32 nm colloidal silica material, stabilized at pH 8.6, was obtained from the Institute for Reference Materials and Measurements (IRMM), European Commission Joint Research Centre, Geel, Belgium) was 83 ± 21%. The SANCO/10684/2009 document concerning method validation and quality control procedures for pesticide residue analysis in food and feed states that the recovery should be between 70 and 120% and that the within lab reproducibility of a method should not exceed 20% [45]. This means that the recovery is acceptable while the reproducibility is on the limit. This however, was considered acceptable since the SANCO document is applicable to well-established methods for well-defined analytes such as pesticides and not to less well-defined particulate materials as in this study.

### Blood biochemistry determination

In plasma, taken after 28-days of exposure, alanine aminotransferase (ALT), aspartate aminotransferase (AST) and alkaline phosphatase (ALP) activity, creatinin, total protein and urea, were determined using standard kits (Beckman-Coulter, Woerden, the Netherlands). In plasma, taken after 84-days of exposure, the same biochemical markers as for the 28-day exposure were determined, as well as lactate dehydrogenase (LDH) activity, uric acid, Zn, Fe, HDL-, LDL- and total cholesterol, triglycerides, and glucose levels using standard kits (Beckman-Coulter). ALT, ASP, ALP, creatinin, total protein, urea, LDH, uric acid, Zn, Fe, HDL, LDL and total cholesterol, triglycerides and glucose were determined with a clinical autoanalyzer (LX20-Pro, Beckman-Coulter) using standard kits which have been developed for this system.

### Immunotoxicity on mesenteric lymph nodes and spleen

The excised mesenteric lymph nodes (MLN) and approximately one third of the spleen of each rat were stored separately in Iscove’s modified Dulbecco’s medium (IMDM; Gibco, Grand Island, NY) on ice. The organs were pressed gently through a cell strainer (70-μm nylon; Falcon, Becton-Dickinson Labware, Franklin Lakes, NJ) and the cells were suspended in 25 ml IMDM, supplemented with 10% fetal calf serum (FCS; PAA, Linz, Austria), 100 IU/ml penicillin, and 100 μg/ml streptomycin, referred to as complete Iscove’s medium. Next, the cell suspensions were centrifuged at 300g for 10 min (4°C) and the pellets were resuspended in 20 ml complete Iscove’s medium. Finally, cells were counted using a Coulter Counter (Coulter Electronics, Luton, UK). For the lymphocyte transformation test, 4*10^5^ cells, isolated from the MLN or spleen, were cultured in six-fold in 150 μl complete Iscove’s medium in U-bottom 96-well microtiter plates (Greiner, Frickenhausen, Germany). In three of the six wells of the first Plate 15 μg/ml LPS (final concentration) was present (B-cell proliferation). In parallel, in three of the six wells of the second Plate 5 μg/ml Con A (final concentration) was present (T-cell proliferation). After 24 h (LPS) or 48 h (Con A) incubation in a humidified atmosphere containing 5% CO_2_ at 37°C, 37 kBq [methyl-3H]thymidine ([3H]TdR; Amersham, Little Chalfont, UK) was added to the wells. Cells were incubated for another 24 h followed by harvesting of the cells onto glass-fibre filters (LKB-Wallac, Espoo, Finland) using a multiple cell culture harvester (LKB-Wallac). Radioactivity was counted using an LKB Wallac 1205 Betaplate Beta Liquid Scintillation Counter.

In addition, natural killer (NK) cell activity was evaluated in cells from spleen cell suspensions by overnight incubation at 37°C as described elsewhere [46]. The activity of NK cells was measured as the ability of 2*10^6^ spleen cells to lyse 1*10^4^^51^Cr-labelled YAC-1 target cells during a 4 h co-incubation in 96-well cell culture plates (Greiner) at 37°C. Radioactivity was counted using a Perkin-Elmer Packard Cobra II Auto Gamma Counter. NK-cell activity was given as a percentage of the maximal release by YAC cells, calculated as (radioactivity counts in the supernatant minus spontaneous release by YAC)/(maximal release by YAC cells minus the spontaneous release by YAC cells).

### Plasma IgG and IgM levels

Plasma IgG and IgM levels were measured using rat IgG and rat IgM ELISA kits (E25G and E25M, respecively; ICL, Gentaur, the Netherlands). IgG was measured at 20,000-, 40,000-, and 80,000-fold serum dilutions, while IgM was measured at a 600-fold serum dilution.

### Cytokine levels in culture supernatants

MLN and spleen cells were incubated with LPS and Con A using the same cell concentrations and LPS and Con A concentrations as described above. Incubation with LPS was for 48 h, while incubation with Con A was for 72 h. To determine the cytokine levels in the culture supernatants a 4-plex panel (Bio-Rad, Hercules, CA) was used in case of LPS-stimulation (IL-1β, IL-6, IL-10, and TNF-α), while a 9-plex panel (Bio-Rad) was used in case of Con A-stimulation (IFN-γ, IL-1β, IL-2, IL-4, IL-6, IL-10, IL-13, IL-17α, and TNF-α). A volume of 100 μl Bio-Plex assay buffer (Bio-Rad) was added to 96-wells filter bottom plates (Bio-Rad) to pre-wet the plate. Buffer was removed by vacuum after each incubation or wash step. Beads were diluted in assay buffer, and 50 μl/well was added. Then, the plates were washed 2 times with 100 μl Bio-Plex wash buffer (Bio-Rad). Dilution series of the cytokine standards were made ranging from 32,000 to 0.18 pg/ml. Fifty μl of the standards and cell culture supernatants was added to the wells and the plates were vortexed at 1100 rpm for 30 s and incubated at RT for 30 min while vortexing at 300 rpm. After incubation the plates were washed 3 times with 100 μl wash buffer. Detection antibody was diluted in detection antibody diluent (Bio-Rad), and 25 μl/well was added. The plates were again vortexed at 1100 rpm for 30 s, incubated at RT for 30 min while vortexing at 300 rpm and washed 3 times with 100 μl assay buffer. Next, streptavidin-PE was diluted in assay buffer and 50 μl/well was added. The plates were incubated for 10 min at RT. After 3 times washing with 100 μl wash buffer the beads were resuspended in 125 μl assay buffer and read on a Bio-Plex (Bio-Rad). Results were obtained at low photomultiplier tube settings.

### Histological analysis

For histopathology, liver, jejunum, kidney and spleen samples were harvested and fixed in 10% neutral buffered formalin. Subsequently, they were dehydrated in a series of ethanol and embedded in paraffin. Approximately 4 μm thick sections were cut, mounted on glass slides and stained with hematoxylin and eosin (H&E). The sections were observed under an optical microscope (Olympus, BX 60, Japan) at different magnifications. All quantitative histological analyses were performed blind to the treatment of the groups.

For livers, 10 images (each of 1 mm^2^) in 10 slides (3 μm apart) in one of the liver lobes per animal were evaluated. Per slide the total number of inflammatory foci, and the total number of inflammatory cells in these foci was scored. Only foci consisting of more than 10 inflammatory cells were included in the analysis. Furthermore, the total number of apoptotic cells was scored per slide, as well as the presence of necrosis or fibrosis. Necrosis resulted in a positive or negative score, leading to a minimum score of 0 positive slides and a maximum score of 10 positive slides per rat, whereas fibrosis was also indexed on severity per slide (0,=not remarkable, 1 = very mild, 2 = mild, 3 = moderate, 4 = severe, 5 = very severe). Apoptotic cells were also visualized using an Apoptaq® Peroxidase *in situ* apoptosis detection kit (Millipore Corporation, Billerica, USA). Representative micrographs were recorded using a Leica DFC 450 camera (Leica, The Netherlands) fitted onto the microscope.

In jejunum samples, 5 μm transverse sections were cut. The villus height, crypt depth, and villus:crypt ratio were measured using an image-analyzing software package (Cell^D; Olympus Soft Imaging Solutions GmbH, Germany), coupled to an optical microscope (Olympus, Japan). A minimum of 10 villi was measured in one slide per animal. All villus height having lamina propria were measured from the villus tip to the end of the base, except the crypt. Crypt depth measurements were taken from the valley between individual villi to the basolateral membrane.

For SEM-EDX analysis of liver tissue, tissues were fixed and dehydrated as described above, but sections were mounted on silicium free Thermanox^TM^ coverslips (Nunc, Germany). Sections were H&E stained and observed under an optical microscope as described above and then deparaffinized in xylene overnight. The sections were subsequently sputter coated with chromium using a K575X turbo sputter coater (Emitech) and analyzed with SEM-EDX as described before. EDX-mapping analysis was used to search for clusters with increased silica content in the tissue. Here, a detection limit of 100 mg silica/kg tissue was estimated. Liver macrophages, selected with light microscopy, were analyzed individually for the presence of silica in the cells with SEM-EDX.

### Statistical analysis

Results were statistically analyzed using Prism (v5; GraphPad Software, Inc., La Jolla, USA) and GenStat 15^th^ edition (version 15.2.0.8821) software. Body- and organ weights, ICP-MS, cytokine release, gene expression of individual genes, and biochemical analysis results were analyzed with a two-way ANOVA with a Bonferroni post-test. HDC ICP-MS, lymphocyte transformation, NK-activity, and antibody release results were analyzed with a one-way ANOVA with a Bonferroni post-test. Outliers in the (HDC) ICP-MS results were removed according to Chauvenet’s criterion. All histopathology data was analyzed with a logarithmic regression analysis using a Poisson distribution, except for the necrosis and fibrosis data, which were analyzed by logistic regression using a binomial distribution between 0 and 10. For all statistical results, a p-value of ≤ 0.05 was considered significant.

### Transcriptomic analysis

To study the effects of the treatment on the transcriptome of liver cells, a piece of liver tissue was immediately frozen in liquid nitrogen during dissection after the 28-, or 84-day exposure and stored until further use. 1250 μl Trizol and 10–15 zirconia/silica beads (Lab Services BV, Breda, The Netherlands) were added to the tissue after which the tissue was homogenized (homogenizer: Precellys 24, Amsterdam, The Netherlands) at 6500 bpm for 2x 15 s with a 30 s interval. The mixture was centrifuged at 12,000 g for 15 min at 4°C. The supernatant was mixed with 300 μl chloroform, incubated at room temperature for 3 min and centrifuged at 12,000 g for 15 min at 4°C. The aqueous phase was transferred to be mixed with 750 μl isopropyl alcohol, which precipitates total RNA. After overnight incubation at −20°C and centrifuging (20 min, 12,000g at 4°C), the pellet was washed with 75% ethanol, centrifuged again at 12,000g for 10 min at 4°C, and resuspended in RNase-free water. Subsequently, RNA was further purified using the RNeasy Mini Kit (Qiagen, Venlo, The Netherlands). Purity, and concentration of the RNA were assessed using the nanodrop (Isogen, De Meern, The Netherlands) at wavelengths of 230, 260, and 280 nm and RNA integrity was checked on an Agilent 2100 Bioanalyzer (Agilent Technologies, Amsterdam, The Netherlands) with 6000 Nano Chips. RNA was judged as suitable only if samples showed intact bands of 18S and 28S ribosomal RNA subunits, displayed no chromosomal peaks or RNA degradation products, and had a RNA integrity number (RIN) above 8.0.

For each individual rat, total RNA (100 ng) of the liver was labeled using the Ambion WT expression kit (Life Technologies, Bleiswijk, The Netherlands). RNA samples were hybridized on Affymetrix GeneChip Rat Gene 1.1 ST arrays. Hybridization, washing, staining and scanning was performed on an Affymetrix GeneTitan instrument. Array data were analyzed using an in-house, on-line system [47]. Shortly, probesets were redefined according to Dai *et al.*[48] using remapped CDF version 15.1 based on the Entrez Gene database and a robust multi-array (RMA) analysis was used to obtain expression values [49,50]. Gene expression data from one rat in the control and NM-202 medium group did not pass the quality control criteria and were excluded.

Spot intensities were floored to 17, which was followed by 2 log mean- centering and calculation of 2 log ratios of treatments *versus* the average of the control samples. Hierarchical clustering was done using the programs Cluster 3.0 (uncentered correlation; average linkage clustering) and Treeview 1.6 (Eisen Lab, USA). Gene set enrichment analysis (GSEA) was performed to discover the differential expression of biologically relevant sets of genes [51]. For this, several genes sets related to common liver processes were composed using the text-mining tool Anni 2.0 (http://www.biosemantics.org/anni) [52]. Furthermore, several gene sets related to specific toxicological responses in the liver, composed from literature and/or in-house data were used, as well as publicly available gene sets (Additional file 1: Table S12). Significantly enriched gene sets were selected on the basis of a p-value <0.01 in combination with an FDR-value <0.25 according to GSEA statistics. Genes that contributed to the enrichment of these sets were selected and filtered on >1.2x up- or down-regulated *versus* the average of the controls in ≥ 3 out of the 5 rats.

## Abbreviations

SAS: Nanostructured Synthetic Amorphous Silica; LOAEL: Lowest-observed-adverse-effect level; (HDC) ICP-MS: (Hydrodynamic chromatography) inductively coupled plasma mass spectroscopy; MLN: Mesenteric lymph nodes; LPS: Lipopolysaccharide; Con A: Concanavalin A; NK-cells: Natural killer cells; OECD: Organisation for Economic Cooperation and Development; SEM: Scanning electron microscopy; EDX: Energy dispersive X-ray spectroscopy; XPS: X-ray photoelectron spectroscopy; FTIR: Fourier transform infrared spectroscopy; G’: Storage modulus; G”: Loss modulus; ALP: Alkaline phosphatase; ALT: Alanine transaminase; AST: Aspartate transaminase; LDH: Lactate dehydrogenase; GSEA: Gene set enrichment analysis.

## Competing interests

The authors declare that they have no competing interests.

## Authors’ contributions

MvdZ, HB, PJH and RJBP conceived, designed and supervised the experiments, analyzed the data and wrote the manuscript. HJPM, RLAPH and AACMP contributed to manuscript preparation. MvdZ, MJG, EK, ZHR, and JSO performed experiments. RJV and ERG performed the immunotoxicity analysis. PT performed the SEM-EDX analysis. KR provided NM-202 and assisted in the characterization of pristine materials. All authors read and approved the final manuscript.

## Supplementary Material

Additional file 1 **Intended and actual silica exposure doses (Table S1).** Silica (in the nano-size range) content in large intestinal contents after 28-days of exposure **(Table S2)**; Body and organ weights after 28-, or 84-days of exposure **(Table S3-4)**; Cytokine production by proliferating B- and T-cells, isolated from the spleen and MLN after 28-, or 84-days of exposure **(Table S5-8)**; Incidence and severity of fibrosis in the liver of animals exposed to SAS or NM-202 for 84 days **(Table S9)**; Gene expression in liver of animals treated with SAS or NM-202 for 28 or 84 days **(Table S10)**; Composition of the juices for the *in vitro* digestion model **(Table S11)**; Gene sets used for gene set enrichment analysis **(Table S12)**; SEM-EDX characterization of SAS and NM-202 in the feed matrix before and after digestion *in vitro***(Figure S1)**; Systemic and immunotoxic responses in SAS and NM-202 treated animals **(Figure S2)**; Quantitative histopathological evaluation of jejunum from animals treated with SAS or NM-202 for 28 days **(Figure S3)**; Methods, XPS characterization. Click here for file
