# Supplementary material for: Sub-chronic toxicity study in rats orally exposed to nanostructured silica
Source: Part Fibre Toxicol. 2014 Feb 7;11:8. doi: 10.1186/1743-8977-11-8 (PMC3922429; doi:10.1186/1743-8977-11-8)
Supplement: Additional file 1 — Intended and actual silica exposure doses (Table S1). Silica (in the nano-size range) content in large intestinal contents after 28-days of exposure (Table S2); Body and organ weights after 28-, or 84-days of exposure (Table S3-4); Cytokine production by proliferating B- and T-cells, isolated from the spleen and MLN after 28-, or 84-days of exposure (Table S5-8); Incidence and severity of fibrosis in the liver of animals exposed to SAS or NM-202 for 84 days (Table S9); Gene expression in liver of animals treated with SAS or NM-202 for 28 or 84 days (Table S10); Composition of the juices for the in vitro digestion model (Table S11); Gene sets used for gene set enrichment analysis (Table S12); SEM-EDX characterization of SAS and NM-202 in the feed matrix before and after digestion in vitro(Figure S1); Systemic and immunotoxic responses in SAS and NM-202 treated animals (Figure S2); Quantitative histopathological evaluation of jejunum from animals treated with SAS or NM-202 for 28 days (Figure S3); Methods, XPS characterization. [file 1743-8977-11-8-S1.docx]

**ADDITIONAL FILE 1**

Sub-chronic toxicity study in rats orally exposed to nanostructured silica

*Meike van der Zande^1*^, Rob J. Vandebriel^2^, Maria J. Groot^1^, Evelien Kramer^1^, Zahira Herrera Rivera^1^, Kirsten Rasmussen^3^, Jan S. Ossenkoppele^1^, Peter Tromp^4^, Eric R. Gremmer^2^, Ruud J. B. Peters^1^, Peter J. Hendriksen^1^, Hans J.P. Marvin^1^, Ron L.A.P. Hoogenboom^1^, Ad A.C.M. Peijnenburg^1^, and Hans Bouwmeester^1*^*

^1^RIKILT – Wageningen University & Research Centre, 6700 AE Wageningen, The Netherlands.

^2^National Institute for Public Health and the Environment, 3720 BA Bilthoven, The Netherlands

^3^Joint Research Centre, 21027 Ispra (VA), Italy

^4^TNO Earth, Environmental and Life Sciences, 3508 TA Utrecht, The Netherlands

* Corresponding Authors: [meike.vanderzande@wur.nl](mailto:meike.vanderzande@wur.nl); [hans.bouwmeester@wur.nl](mailto:hans.bouwmeester@wur.nl)

**Table S1**: Intended and actual silica exposure doses.

| **Group** | **Intended exposure dose of total silica**  **(mg/kg bw/day)** | **Actual exposure dose** | | | | |
| --- | --- | --- | --- | --- | --- | --- |
|  |  | **Dose of total silica**  **from SAS/NM-202/ control feed mixture**  **(mg/kg bw/day)** | **Dose of total silica**  **from standard diet (mg/kg bw/day)** | **Dose of total silica from drinking water (mg/kg bw/day)** | **Total silica**  **(mg/kg bw/day)** | **Silica in the nano-size range (mg/kg bw/day)** |
| SAS low | 100 | 83 (~0.36 g mixture/rat/day) | 137 (~26.64 g diet/rat/day) | 2.4 (~45 ml/rat/day) | 222 | 33 |
| SAS medium | 1000 | 819 (~3.56 g mixture/rat/day) | 121 (~23.44 g diet/rat/day) | 2.4 (~45 ml/rat/day) | 942 | 328 |
| SAS high | 2500 | 2047 (~8.90 g mixture/rat/day) | 93 (~18.10 g diet/rat/day) | 2.4 (~45 ml/rat/day) | 2142 | 819 |
| NM-202 low | 100 | 82 (~0.36 g mixture/rat/day) | 137 (~26.64 g diet/rat/day) | 2.4 (~45 ml/rat/day) | 221 | 82 |
| NM-202 medium | 500 | 405 (~1.78 g mixture/rat/day) | 130 (~25.22 g diet/rat/day) | 2.4 (~45 ml/rat/day) | 537 | 405 |
| NM-202 high | 1000 | 810 (~3.56 g mixture/rat/day) | 121 (~23.44 g diet/rat/day) | 2.4 (~45 ml/rat/day) | 933 | 810 |
| Negative control | 0 | 10 (~3.56 g mixture/rat/day) | 121 (~23.44 g diet/rat/day) | 2.4 (~45 ml/rat/day) | 133 | <21 |

One mixture of SAS, and NM-202 in feed (*i.e.* standard diet and chocolate milk) was prepared. Dosing was based on the weight of the animals, and higher dosed animals were offered more of this feed mixture than lower dosed animals. The actual exposure doses were calculated from the (HDC) ICP-MS silicon measurements in the SAS/NM-202/control feed mixtures, standard feed pellets, and drinking water. Results from these measurements were: 80.5 ± 20.9 mg silica/g feed mixture for SAS, 79.6 ± 20.7 mg silica/g feed mixture for NM-202, 0.95 ± 0.19 mg silica/g feed mixture for the control, and 1.8 ± 0.9 mg silica/g feed in the standard feed pellets. The fractions of silica in the nano-size range were ~40 wt% for SAS and ~100 wt% for NM-202. Exposure to silica originating from consumption of standard feed pellets and drinking water was based on an average feed intake of 27 g/day [1] and an average water intake of 45 ml/day [1] for the measured average body weight of 350 g. The daily intake of the feed mixture and standard diet thereafter, during the entire study is given for each exposure group.

1. EPA US: **Recommendations for and Documentation of Biological Values for Use in Risk Assessment**. 1988, EPA/600/6-87/008.

**Table S2**: **S**ilica (in the nano-size range) content in large intestinal contents in mg silica (in the nano-size range)/kg content (mean ± SEM, n=5) after 28-day exposure.

|  | **Large intestinal contents** | | | |
| --- | --- | --- | --- | --- |
|  | **Total silica (mg/kg content) mean ± SEM** | **Silica in the nano-size range  (mg/kg content) mean ± SEM** | **Content of silica in the nano-size range as a percentage of the total silica content** |  |
| Control | 4878 ± 341 | 1234 ± 161 | 25 |  |
| SAS low | 4942 ± 404 | 2700 ± 302 | 55 |  |
| SAS medium | 7831 ± 844 | 8300 ± 1138*^a^* | 106*^a^* |  |
| SAS high | 11596 ± 1194*^a^* | 6240 ± 1088*^a^* | 54 |  |
| NM-202 low | 5477 ± 476 | 5340 ± 1305*^a^* | 97*^a^* |  |
| NM-202 medium | 7221 ± 527 | 5867 ± 617*^a^* | 81*^a^* |  |
| NM-202 high | 6205 ± 715 | 5000 ± 688 | 81*^a^* |  |

*^a^* Significant difference *versus* control (p<0.05). SEM: standard error of the mean.

**Table S3**: Body and organ weights in grams (mean ± SEM, n=5) after 28-days of exposure.

|  | **Control** | **SAS low** | **SAS medium** | **SAS high** | **NM-202 low** | **NM-202 medium** | **NM-202 high** |
| --- | --- | --- | --- | --- | --- | --- | --- |
| Body | 375.52 ± 6.14 | 371.90 ± 6.93 | 366.14 ± 11.15 | 372.30 ± 6.62 | 359.56 ± 9.83 | 371.70 ± 7.21 | 374 ± 6.44 |
| Liver | 14.28 ± 0.24 | 14.25 ± 0.40 | 13.93 ± 0.74 | 13.81 ± 0.51 | 13.68 ± 0.41 | 14.65 ± 0.73 | 14.39 ± 0.45 |
| Testis | 3.69 ± 0.09 | 3.79 ± 0.18 | 3.83 ± 0.09 | 3.84 ± 0.06 | 3.73 ± 0.11 | 3.91 ± 0.10 | 3.79 ± 0.03 |
| Kidney | 2.64 ± 0.09 | 2.48 ± 0.09 | 2.44 ± 012 | 2.53 ± 0.08 | 2.51 ± 0.11 | 2.51 ± 005 | 2.54 ± 0.03 |
| Brain | 1.88 ± 0.03 | 1.68 ± 0.13 | 1.89 ± 0.02 | 1.86 ± 0.04 | 1.82 ± 0.02 | 1.82 ± 0.02 | 1.80 ± 0.05 |
| Lung | 1.69 ± 0.08 | 1.62 ± 0.08 | 1.54 ± 0.06 | 1.57 ± 0.05 | 1.31 ± 005 | 1.73 ± 0.05 | 1.52 ± 0.07 |
| Heart | 1.40 ± 0.03 | 1.40 ± 0.02 | 1.36 ± 0.08 | 1.43 ± 0.04 | 1.34 ± 0.03 | 1.34 ± 0.09 | 1.40 ± 0.03 |
| Thymus | 0.74 ± 0.06 | 0.71 ± 0.08 | 0.61 ± 0.03 | 0.69 ± 0.04 | 0.62 ± 0.05 | 0.73 ± 0.04 | 0.61 ± 0.04 |
| Spleen | 0.86 ± 0.04 | 0.84 ± 0.03 | 0.79 ± 0.02 | 0.86 ± 0.03 | 0.80 ± 0.03 | 0.88 ± 0.03 | 0.80 ± 0.05 |

SEM: standard error of the mean

**Table S4:** Body and organ weights in grams (mean ± SEM, n=5) at the last exposure after 84-days of exposure.

|  | **SAS high** | **NM-202 high** | **Control** |
| --- | --- | --- | --- |
| Body | 437.40 ± 40.00 | 420.46 ± 10.20 | 434.84 ± 7.69 |
| Liver | 14.47 ± 0.49 | 14.26 ± 0.70 | 14.12 ± 0.33 |
| Testis | 4.20 ± 0.14 | 4.05 ± 0.10 | 4.07 ± 0.08 |
| Kidney | 2.96 ± 013 | 2.82 ± 0.09 | 2.74 ± 0.05 |
| Brain | 2.04 ± 0.09 | 1.94 ± 0.03 | 1.95 ± 0.02 |
| Lung | 1.71 ± 0.15 | 1.55 ± 006 | 1.58 ± 0.04 |
| Heart | 1.61 ± 0.13 | 1.52 ± 0.07 | 1.50 ± 0.05 |
| Thymus | 0.56 ± 0.11 | 0.44 ± 004 | 0.46 ± 0.03 |
| Spleen | 0.91 ± 0.13 | 0.79 ± 0.01 | 0.77 ± 0.03 |

SEM: standard error of the mean.

**Table S5:** Cytokine production by proliferating B-cells in pg/mL (mean ± SEM, n=5), isolated from spleen and MLN after 28-days of exposure.

|  | **Control** | **SAS low** | **SAS medium** | **SAS high** | **NM-202 low** | **NM-202 medium** | **NM-202 high** | | |
| --- | --- | --- | --- | --- | --- | --- | --- | --- | --- |
|  | *Cells isolated from spleen; LPS treated* | | | | | | | |  |
| IL-1β | 4641 ± 282 | 5065 ± 269 | 4672 ± 440 | 4897 ± 84 | 4476 ± 176 | 4412 ± 279 | 4990 ± 198 | | |
| IL-6 | 10776 ± 1101 | 11467 ± 529 | 9359 ± 840 | 11327 ± 837 | 9779 ± 491 | 9692 ± 878 | 11184 ± 814 | | |
| IL-10 | 28424 ± 1274 | 27167 ± 1497 | 30565 ± 2817 | 29687 ± 871 | 30194 ± 1888 | 29136 ± 1526 | 31613 ± 533 | | |
| TNFα | 5471 ± 341 | 6407 ± 219 | 5262 ± 382 | 6480 ± 160 | 5344 ± 307 | 5368 ± 261 | 5689 ± 150 | | |
|  | *Cells isolated from MLN; LPS treated* | | | | | | |  |  |
| IL-1β | 2396 ± 115 | 2403 ± 177 | 2181 ± 69 | 2400 ± 88 | 2276 ± 254 | 2384 ± 281 | 2301 ± 167 | | |
| IL-6 | 6278 ± 569 | 6128 ± 614 | 5047 ± 424 | 5931 ± 590 | 5564 ± 320 | 6061 ± 953 | 5499 ± 220 | | |
| IL-10 | 15219 ± 1035 | 15942 ± 911 | 14132 ± 616 | 13408 ± 245 | 15133 ± 1073 | 16566 ± 1847 | 14573 ± 384 | | |
| TNFα | 1881 ± 109 | 1993 ± 128 | 1660 ± 120 | 2027 ± 182 | 1800 ± 102 | 1815 ± 154 | 1748 ± 32 | | |

Abbreviations: SEM: standard error of the mean, LPS: lipopolysaccharides.

**Table S6:** Cytokine production by proliferating T-cells in pg/mL (mean ± SEM, n=5), isolated from spleen and MLN after 28-days of exposure.

|  | **Control** | **SAS low** | **SAS medium** | **SAS high** | **NM-202 low** | **NM-202 medium** | **NM-202 high** |
| --- | --- | --- | --- | --- | --- | --- | --- |
|  | *Cells isolated from spleen; ConA treated* | | | | | | |
| IL-1β | 241 ± 40 | 281 ± 45 | 187 ± 36 | 280 ± 19 | 222 ± 20 | 189 ± 19 | 231 ± 24 |
| IL-6 | 163 ± 34 | 176 ± 41 | 139 ± 18 | 191 ± 39 | 154 ± 18 | 141 ± 7 | 178 ±21 |
| IL-10 | 5585 ± 793 | 6331 ± 400 | 6218 ± 1148 | 6689 ± 502 | 7835 ± 275*^a^* | 5583 ± 424 | 6694 ± 391 |
| TNFα | 1669 ± 170 | 1865 ± 263 | 1792 ± 313 | 1814 ± 98 | 1635 ± 124 | 1671 ± 97 | 1612 ± 75 |
| IFNɣ | 3235 ± 129 | 4274 ± 455 | 4087 ± 543 | 3853 ± 338 | 3817 ± 310 | 4019 ± 639 | 3659 ± 294 |
| IL-2 | 4176 ± 385 | 4752 ± 461 | 4548 ± 412 | 4886 ± 148 | 4352 ± 145 | 4397 ± 256 | 4984 ± 401 |
| IL-4 | 30 ± 6 | 24 ± 4 | 24 ± 6 | 22 ± 3 | 28 ± 3 | 27 ± 3 | 28 ± 5 |
| IL-13 | 12 ± 3 | 13 ± 3 | 11 ± 4 | 10 ± 3 | 11 ± 3 | 13 ± 3 | 15 ± 4 |
| IL-17α | 1637 ± 585 | 1618 ± 278 | 1589 ± 338 | 2010 ± 300 | 2500 ± 94 | 1608 ± 318 | 2268 ± 318 |
|  | *Cells isolated from MLN; ConA treated* | | | | | | |
| IL-1β | 81 ± 12 | 80 ± 9 | 81 ± 16 | 72 ± 10 | 69 ± 22 | 93 ± 23 | 70 ± 18 |
| IL-6 | 119 ± 14 | 150 ± 17 | 118 ± 16 | 72 ± 11 | 122 ± 28 | 174 ± 18 | 111 ± 13 |
| IL-10 | 3732 ± 103 | 4242 ± 216 | 3690 ± 351 | 3261 ± 117 | 3407 ± 171 | 4484 ± 830 | 3825 ± 356 |
| TNFα | 1057 ± 25 | 1099 ± 84 | 1074 ± 72 | 947 ± 59 | 1066 ± 136 | 1083 ± 96 | 972 ± 92 |
| IFNɣ | 2767 ± 208 | 3296 ± 125 | 2563 ± 92 | 2805 ± 162 | 2710 ± 335 | 3487 ± 626 | 2270 ± 244 |
| IL-2 | 1875 ± 280 | 2107 ± 113 | 1902 ± 133 | 1608 ± 240 | 1783 ± 194 | 1745 ± 135 | 1706 ± 153 |
| IL-4 | 39 ± 6 | 26 ± 2 | 28 ± 2 | 24 ± 2 | 31 ± 5 | 30 ± 3 | 32 ± 4 |
| IL-13 | ND | ND | ND | ND | ND | ND | ND |
| IL-17α | 287 ± 51 | 289 ± 30 | 335 ± 77 | 193 ± 17 | 235 ± 46 | 362 ± 74 | 340 ± 97 |

*^a^* Significant difference *versus* the control group (p<0.05). Abbreviations: SEM: standard error of the mean, ConA: concanavalin A.

**Table S7:** Cytokine production by proliferating B-cells in pg/mL (mean ± SEM, n=5), isolated from spleen and MLN after 84 days of exposure.

|  | **SAS high** | **NM-202 high** | **control** |
| --- | --- | --- | --- |
|  | *Cells isolated from spleen; LPS treated* | | |
| IL-1β | 4525 ± 266 | 4616 ± 104 | 4959 ± 206 |
| IL-6 | 9586 ± 623 | 9821 ± 419 | 10587 ± 538 |
| IL-10 | 28247 ± 620*^a^* | 31450 ± 1834 | 32057 ± 613 |
| TNFα | 6052 ± 297 | 6101 ± 312 | 6517 ± 538 |
|  | *Cells isolated from MLN; LPS treated* | | |
| IL-1β | 2120 ± 49 | 2047 ± 127 | 1865 ± 62 |
| IL-6 | 5365 ± 245 | 5144 ± 287 | 5525 ± 450 |
| IL-10 | 16369 ± 771*^b^* | 17451 ± 864*^b^* | 16056 ± 293 |
| TNFα | 1706 ± 102 | 1605 ± 58 | 1754 ± 189 |

*^a^* Significant difference *versus* the control and *^b^* significant increase *versus* the same treatment group after 28-days of exposure (p<0.05). Abbreviations: SEM: standard error of the mean, LPS: lipopolysaccharides.

**Table S8**: Cytokine production by proliferating T-cells in pg/mL (mean ± SEM, n=5), isolated from spleen and MLN after 84 days of exposure.

|  | **SAS high** | **NM-202 high** | **Control** | |
| --- | --- | --- | --- | --- |
|  | *Cells isolated from spleen; ConA treated* | | |  |
| IL-1β | 265 ± 8 | 302 ± 26 | 271 ± 34 | |
| IL-6 | 209 ± 22 | 210 ± 13 | 212 ± 26 | |
| IL-10 | 7860 ± 569*^a^* | 9325 ± 631 | 9369 ± 1193 | |
| TNFα | 2166 ± 223 | 2167 ± 176 | 2310 ± 140 | |
| IFNɣ | 3313 ± 251 | 3628 ± 437 | 2909 ± 131 | |
| IL-2 | 5065 ± 691 | 5062 ± 678 | 5630 ± 483 | |
| IL-4 | 27 ± 7 | 25 ± 4 | 29 ± 4 | |
| IL-13 | 9 ± 4 | 19 ± 5 | 23 ± 10 | |
| IL-17α | 2785 ± 98 | 2920 ± 131 | 2296 ± 346 | |
|  | *Cells isolated from MLN; ConA treated* | | |  |
| IL-1β | 77 ± 8 | 86 ± 8 | 74 ± 12 | |
| IL-6 | 51 ± 11 | 87 ± 7 | 68 ± 12 | |
| IL-10 | 2783 ± 265 | 3036 ± 272*^b^* | 2792 ± 253*^b^* | |
| TNFα | 1230 ± 39 | 1171 ± 113 | 1211 ± 114 | |
| IFNɣ | 2735 ± 121 | 2610 ± 286 | 2447 ± 266 | |
| IL-2 | 2572 ± 314 | 2438 ± 277 | 2356 ± 265*^b^* | |
| IL-4 | 27 ± 4 | 26 ± 3 | 23 ± 2 | |
| IL-13 | ND | ND | ND | |
| IL-17α | 197 ± 14 | 264 ± 37 | 207 ± 31 | |

*^a^* Significant difference *versus* the control group and *^b^* significant increase *versus* the same treatment group after 28-days of exposure (p<0.05). Abbreviations: SEM: standard error of the mean, ConA: concanavalin A.

**Table S9:** Incidence and severity of fibrosis in the liver of animals exposed to SAS or NM-202 for 84 days (n=5).

| ***n*** | **control** | **SAS high** | **NM-202 high** |
| --- | --- | --- | --- |
|  | **(incidence/severity)** | **(incidence/severity)** | **(incidence/severity)** |
| 1 | 9/0; 1/1 | 10/0 | 5/0; 3/1; 2/2 |
| 2 | 10/0 | 8/0; 2/1 | 7/0; 3/1 |
| 3 | 8/0; 2/1 | 6/0; 2/1; 2/2 | 7/0; 3/2 |
| 4 | 10/0 | 5/0; 2/1; 3/2 | 5/0; 4/1; 1/2 |
| 5 | 8/0; 1/1; 1/2 | 6/0; 3/1; 1/2 | 6/0; 1/1; 3/2 |

Severity index: 0=not remarkable, 1=very mild, 2=mild, 3=moderate, 4=severe, 5=very severe. Ten slides were evaluated per animal.

**Table S10:** Gene expression (mean ± SEM) in liver of animals treated with SAS or NM-202 for 28 or 84 days.

|  | | **28-day exposure** | | | | | | | | |
| --- | --- | --- | --- | --- | --- | --- | --- | --- | --- | --- |
|  | | **SAS low (n=5)** | | **SAS medium (n=5)** | | **SAS high (n=5)** | | | |  |
| IL-1β | | 38.83 ± 3.24 | | 43.96 ± 6.73*^a^* | | 43.17 ± 5.38*^a^* | | | |  |
| IL-2 | | 12.46 ± 0.32 | | 11.73 ± 0.33 | | 11.88 ± 0.44 | | | |  |
| IL-4 | | 9.53 ± 0.27 | | 9.81 ± 0.26 | | 9.65 ± 0.23 | | | |  |
| IL-6 | | 7.98 ± 0.27 | | 7.70 ± 0.17 | | 7.36 ± 0.34 | | | |  |
| IL-10 | | 8.88 ± 0.33 | | 9.97 ± 0.73 | | 9.38 ± 0.63 | | | |  |
| IL-17α | | 13.65 ± 0.51 | | 15.12 ± 0.79 | | 13.69 ± 0.44 | | | |  |
| IL-18 | | 32.57 ± 0.98 | | 37.34 ± 2.52 | | 33.76 ± 1.56 | | | |  |
| TNF | | 17.07 ± 0.56 | | 15.65 ± 0.58 | | 15.22 ± 0.70 | | | |  |
| INFɣ | | 10.35 ± 0.31 | | 10.51 ± 0.11 | | 10.22 ± 0.24 | | | |  |
|  | **NM-202 low (n=5)** | | **NM-202 medium (n=4)** | | **NM-202 high (n=5)** | | **Control (n=5)** |  |  |  |
| IL-1β | 36.81 ± 3.06 | | 42.97 ± 3.59 | | 37.03 ± 3.76 | | 36.75 ± 2.46 |  |  |  |
| IL-2 | 11.89 ± 0.42 | | 11.80 ± 0.40 | | 12.19 ± 0.40 | | 12.30 ± 0.26 |  |  |  |
| IL-4 | 9.21 ± 0.26 | | 9.59 ± 0.26 | | 9.93 ± 0.47 | | 9.72 ± 0.15 |  |  |  |
| IL-6 | 7.59 ± 0.30 | | 7.98 ± 0.28 | | 7.02 ± 0.18 | | 7.31 ± 0.18 |  |  |  |
| IL-10 | 9.49 ± 0.47 | | 10.38 ± 1.15 | | 9.64 ± 0.65 | | 10.04 ± 0.39 |  |  |  |
| IL-17α | 15.17 ± 0.59 | | 14.36 ± 0.35 | | 14.05 ± 0.57 | | 15.40 ± 0.67 |  |  |  |
| IL-18 | 36.71 ± 2.36 | | 33.15 ± 2.40 | | 33.35 ± 2.59 | | 34.42 ± 1.47 |  |  |  |
| TNF | 15.55 ± 0.49 | | 17.19 ± 0.56 | | 15.97 ± 0.90 | | 16.99 ± 1.09 |  |  |  |
| INFɣ | 10.90 ± 0.45 | | 10.51 ± 0.41 | | 10.65 ± 0.24 | | 9.92 ± 0.27 |  |  |  |
|  | **84-day exposure** | | | | | | | |  |  |
|  | **SAS high (n=5)** | | **NM-202 high (n=5)** | | **Control (n=4)** | | | |  |  |
| IL-1β | 31.72 ± 1.63*^b^* | | 36.71 ± 1.63 | | 34.89 ± 1.50*^b^* | | | |  |  |
| IL-2 | 12.44 ± 0.43 | | 13.30 ± 0.70 | | 12.01 ± 0.64 | | | |  |  |
| IL-4 | 9.90 ± 0.39 | | 9.65 ± 0.45 | | 9.76 ± 0.22 | | | |  |  |
| IL-6 | 7.67 ± 0.21 | | 7.62 ± 0.12 | | 7.80 ± 0.37 | | | |  |  |
| IL-10 | 9.46 ± 0.33 | | 9.62 ± 0.32 | | 9.94 ± 0.43 | | | |  |  |
| IL-17α | 14.23 ± 0.39 | | 14.06 ± 0.30 | | 14.42 ± 0.83 | | | |  |  |
| IL-18 | 27.41 ± 1.05*^b^* | | 31.10 ± 2.06 | | 28.85 ± 0.84 | | | |  |  |
| TNF | 15.83 ± 0.97 | | 16.78 ± 0.16 | | 15.77 ± 0.87 | | | |  |  |
| INFɣ | 10.44 ± 0.22 | | 10.30 ± 0.21 | | 10.45 ± 0.44 | | | |  |  |

Expression values are given as the measured signal intensities on the microarray *^a^* Significant difference *versus* the control group and *^b^* significant increase *versus* the same treatment group after 28-day exposure (p<0.05). Abbreviations: SEM: standard error of the mean.

**Table S11:** Composition of the juices for the *in vitro* digestion model (amounts based on 1000 ml of juice) [1].

|  | **saliva pH 6.8 ± 0.1** | **gastric juice pH 1.3 ± 0.1** | **duodenal juice pH 8.1 ± 0.1** | **bile juice pH 8.2 ± 0.1** |  |
| --- | --- | --- | --- | --- | --- |
| Inorganic constituents | - 896 mg KCl | - 2752 mg NaCl | - 7012 mg NaCl | - 5259 mg NaCl | |
|  | - 200 mg KSCN | - 306 mg NaH_2_PO_4_ · H_2_O | - 3388 mg NaHCO_3_ | - 5785 mg NaHCO_3_ | |
|  | - 1021 mg NaH_2_PO_4_ · H_2_O | - 824 mg KCl | - 80 mg KH_2_PO_4_ | - 376 mg KCl | |
|  | - 570 mg Na_2_SO_4_ | - 6.5 ml 37% HCl | - 564 mg KCl | - 150 ml HCl (37%) | |
|  | - 298 mg NaCl | - 650 mg glucose | - 50 mg MgCl_2_· 6H_2_O |  | |
|  | - 1694 mg NaHCO_3_ | - 20 mg glucuronic acid | - 180 ml HCl (37%) |  | |
|  |  |  |  |  | |
| Organic constituents | - 200 mg urea | - 85 mg urea | - 100 mg urea | - 250 mg urea | |
|  | - 290 mg amylase | - 330 mg glucosaminehydrochloride | - 151 mg CaCl_2_ | - 167.5 mg CaCl_2_ | |
|  | - 15 mg uric acid | - 1 g BSA | - 1 g BSA | - 1.8 g BSA | |
|  | - 25 mg mucin | - 2.5 g pepsin | - 9 g pancreatin | - 30 g bile | |
|  | - Milli-Q water | - 3 g mucin | - 1.5 g lipase | - Milli-Q water | |
|  |  | - Milli-Q water | - Milli-Q water |  | |
|  |  |  |  | **Sodium carbonate solution** | |
|  |  |  |  | - 84.7 g NaHCO_3_ | |
|  |  |  |  | - Milli-Q water | |

1. Versantvoort CHM, Oomen AG, Van de Kamp E, Rompelberg CJM, Sips AJAM: **Applicability of an in vitro digestion model in assessing the bioaccessibility of mycotoxins from food**. *Food Chem Toxicol* 2005, 43:31-40.

**Table S12:** Gene sets used for gene set enrichment analysis (GSEA).

| **Name gene set** | **Source/reference** |
| --- | --- |
| - c2.cp.kegg.v3.1.symbols.gmt | - http://www.broadinstitute.org/gsea |
| - c2.cp.biocarta.v3.1.symbols.gmt | - http://www.broadinstitut e.org/gsea |
| - Common_liver_processes_v1.gmx | - Self-made from reference [[1](#_ENREF_1)] |
| - TOX_Johnson_Johnson_100_Compounds_Liver_Symbolsv4.gmx | - Self-made from reference [[2](#_ENREF_2)] combined with in-house data |
| - TOX_TFS_TARGET_GENES.GENE_ SYMBOLv3.gmx | - Self-made from references [[3-9](#_ENREF_3)] combined with in-house data |
| - c2_genmapp_v2_5_adjPeter.gmx | - Adapted from http://www.broadinstitute.org/gsea |
| - Liver_hormones_GENESETS_ALL_ Studies_Combined_v1 | - Self-made from references [[10-17](#_ENREF_10)] |
| - Tox_Action_symbols.gmx | - Self-made from references [[18-23](#_ENREF_18)] combined with in-house data |
| - Liver_Transcription_Factor_Bound_ Genes.gmx | - Self-made from reference [[24](#_ENREF_24)] |

1. Jelier R, Schuemie MJ, Veldhoven A, Dorssers LC, Jenster G, Kors JA: **Anni 2.0: a multipurpose text-mining tool for the life sciences**. *Genome biology* 2008, **9:**R96.

2. McMillian M, Nie AY, Parker JB, Leone A, Bryant S, Kemmerer M, Herlich J, Liu Y, Yieh L, Bittner A, Liu X, Wan J, Johnson MD: **A gene expression signature for oxidant stress/reactive metabolites in rat liver**. *Biochemical pharmacology* 2004, **68:**2249-61.

3. Hu R, Xu C, Shen G, Jain MR, Khor TO, Gopalkrishnan A, Lin W, Reddy B, Chan JY, Kong AN: **Gene expression profiles induced by cancer chemopreventive isothiocyanate sulforaphane in the liver of C57BL/6J mice and C57BL/6J/Nrf2 (-/-) mice**. *Cancer letters* 2006, **243:**170-92.

4. Hu R, Xu C, Shen G, Jain MR, Khor TO, Gopalkrishnan A, Lin W, Reddy B, Chan JY, Kong AN: **Identification of Nrf2-regulated genes induced by chemopreventive isothiocyanate PEITC by oligonucleotide microarray**. *Life sciences* 2006, **79:**1944-55.

5. Banno T, Gazel A, Blumenberg M: **Pathway-specific profiling identifies the NF-kappa B-dependent tumor necrosis factor alpha-regulated genes in epidermal keratinocytes**. *The Journal of biological chemistry* 2005, **280:**18973-80.

6. Tian B, Nowak DE, Jamaluddin M, Wang S, Brasier AR: **Identification of direct genomic targets downstream of the nuclear factor-kappaB transcription factor mediating tumor necrosis factor signaling**. *The Journal of biological chemistry* 2005, **280:**17435-48.

7. Godefroy N, Bouleau S, Gruel G, Renaud F, Rincheval V, Mignotte B, Tronik-Le Roux D, Vayssiere JL: **Transcriptional repression by p53 promotes a Bcl-2-insensitive and mitochondria-independent pathway of apoptosis**. *Nucleic acids research* 2004, **32:**4480-90.

8. Hanlon PR, Zheng W, Ko AY, Jefcoate CR: **Identification of novel TCDD-regulated genes by microarray analysis**. *Toxicology and applied pharmacology* 2005, **202:**215-28.

9. Wang Z, Malone MH, He H, McColl KS, Distelhorst CW: **Microarray analysis uncovers the induction of the proapoptotic BH3-only protein Bim in multiple models of glucocorticoid-induced apoptosis**. *The Journal of biological chemistry* 2003, **278:**23861-7.

10. Henriquez-Hernandez LA, Flores-Morales A, Santana-Farre R, Axelson M, Nilsson P, Norstedt G, Fernandez-Perez L: **Role of pituitary hormones on 17alpha-ethinylestradiol-induced cholestasis in rat**. *The Journal of pharmacology and experimental therapeutics* 2007, **320:**695-705.

11. Horn TL, Torres KE, Naylor JM, Cwik MJ, Detrisac CJ, Kapetanovic IM, Lubet RA, Crowell JA, McCormick DL: **Subchronic toxicity and toxicogenomic evaluation of tamoxifen citrate + bexarotene in female rats**. *Toxicological sciences : an official journal of the Society of Toxicology* 2007, **99:**612-27.

12. Rogers AB, Theve EJ, Feng Y, Fry RC, Taghizadeh K, Clapp KM, Boussahmain C, Cormier KS, Fox JG: **Hepatocellular carcinoma associated with liver-gender disruption in male mice**. *Cancer research* 2007, **67:**11536-46.

13. Silkworth JB, Carlson EA, McCulloch C, Illouz K, Goodwin S, Sutter TR: **Toxicogenomic analysis of gender, chemical, and dose effects in livers of TCDD- or aroclor 1254-exposed rats using a multifactor linear model**. *Toxicological sciences : an official journal of the Society of Toxicology* 2008, **102:**291-309.

14. Feng X, Jiang Y, Meltzer P, Yen PM: **Thyroid hormone regulation of hepatic genes in vivo detected by complementary DNA microarray**. *Molecular endocrinology* 2000, **14:**947-55.

15. Coe KJ, Jia Y, Ho HK, Rademacher P, Bammler TK, Beyer RP, Farin FM, Woodke L, Plymate SR, Fausto N, Nelson SD: **Comparison of the cytotoxicity of the nitroaromatic drug flutamide to its cyano analogue in the hepatocyte cell line TAMH: evidence for complex I inhibition and mitochondrial dysfunction using toxicogenomic screening**. *Chemical research in toxicology* 2007, **20:**1277-90.

16. Dong H, Yauk CL, Williams A, Lee A, Douglas GR, Wade MG: **Hepatic gene expression changes in hypothyroid juvenile mice: characterization of a novel negative thyroid-responsive element**. *Endocrinology* 2007, **148:**3932-40.

17. Hendriksen PJ, Freidig AP, Jonker D, Thissen U, Bogaards JJ, Mumtaz MM, Groten JP, Stierum RH: **Transcriptomics analysis of interactive effects of benzene, trichloroethylene and methyl mercury within binary and ternary mixtures on the liver and kidney following subchronic exposure in the rat**. *Toxicology and applied pharmacology* 2007, **225:**171-88.

18. Steiner G, Suter L, Boess F, Gasser R, de Vera MC, Albertini S, Ruepp S: **Discriminating different classes of toxicants by transcript profiling**. *Environmental health perspectives* 2004, **112:**1236-48.

19. Mandard S, Muller M, Kersten S: **Peroxisome proliferator-activated receptor alpha target genes**. *Cellular and molecular life sciences : CMLS* 2004, **61:**393-416.

20. McMillian M, Nie AY, Parker JB, Leone A, Kemmerer M, Bryant S, Herlich J, Yieh L, Bittner A, Liu X, Wan J, Johnson MD: **Inverse gene expression patterns for macrophage activating hepatotoxicants and peroxisome proliferators in rat liver**. *Biochemical pharmacology* 2004, **67:**2141-65.

21. Nishimura M, Naito S: **Tissue-specific mRNA expression profiles of human phase I metabolizing enzymes except for cytochrome P450 and phase II metabolizing enzymes**. *Drug metabolism and pharmacokinetics* 2006, **21:**357-74.

22. Pierrou S, Broberg P, O'Donnell RA, Pawlowski K, Virtala R, Lindqvist E, Richter A, Wilson SJ, Angco G, Moller S, Bergstrand H, Koopmann W, Wieslander E, Stromstedt PE, Holgate ST, Davies DE, Lund J, Djukanovic R: **Expression of genes involved in oxidative stress responses in airway epithelial cells of smokers with chronic obstructive pulmonary disease**. *American journal of respiratory and critical care medicine* 2007, **175:**577-86.

23. Lodovici M, Luceri C, De Filippo C, Romualdi C, Bambi F, Dolara P: **Smokers and passive smokers gene expression profiles: correlation with the DNA oxidation damage**. *Free radical biology & medicine* 2007, **43:**415-22.

24. Odom DT, Dowell RD, Jacobsen ES, Nekludova L, Rolfe PA, Danford TW, Gifford DK, Fraenkel E, Bell GI, Young RA: **Core transcriptional regulatory circuitry in human hepatocytes**. *Molecular systems biology* 2006, **2:**2006 0017.

**
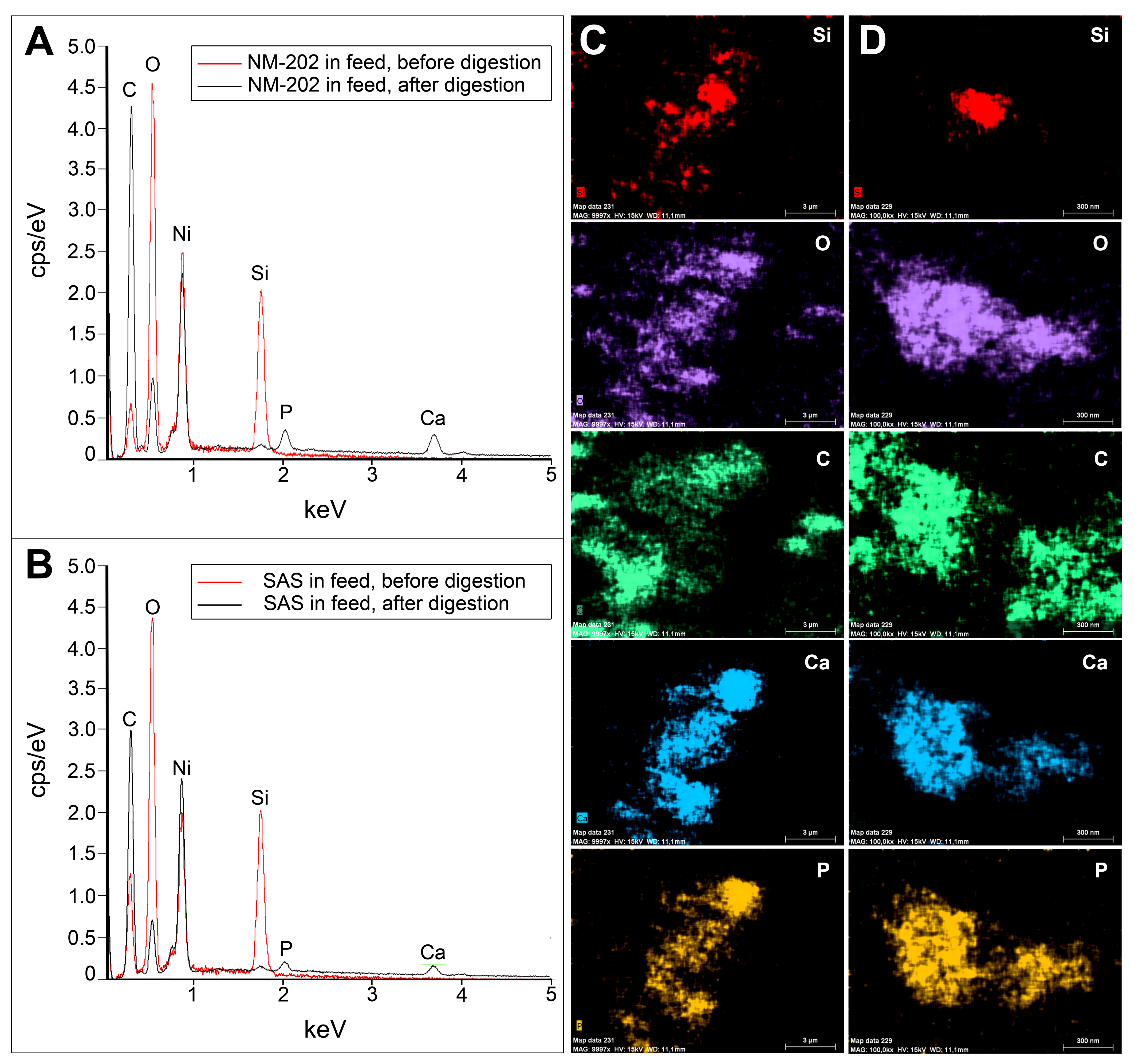
**

**Figure S1**: SEM-EDX characterization of (A, C) NM-202 and (B, D) SAS in the feed matrix before and after digestion *in vitro*. Before digestion, small SiO_2_ particles (*i.e.* <500 nm) were isolated and analyzed, but after digestion no such small particles could be detected and isolated for analysis. Therefore, the materials were washed to remove soluble salts after digestion and larger aggregates (*i.e.* 0.5-10 µm) were analyzed. The smaller peaks representing Si and O (A, B) and the micrographs (C, D) indicate that these aggregates did not consist solely of SiO_2_ particles prohibiting a direct quantitative comparison of the materials before and after digestion. Nevertheless, after digestion, C still appears to be present on both materials, and also Ca and P (likely as CaPO_4_) were present after digestion.

**
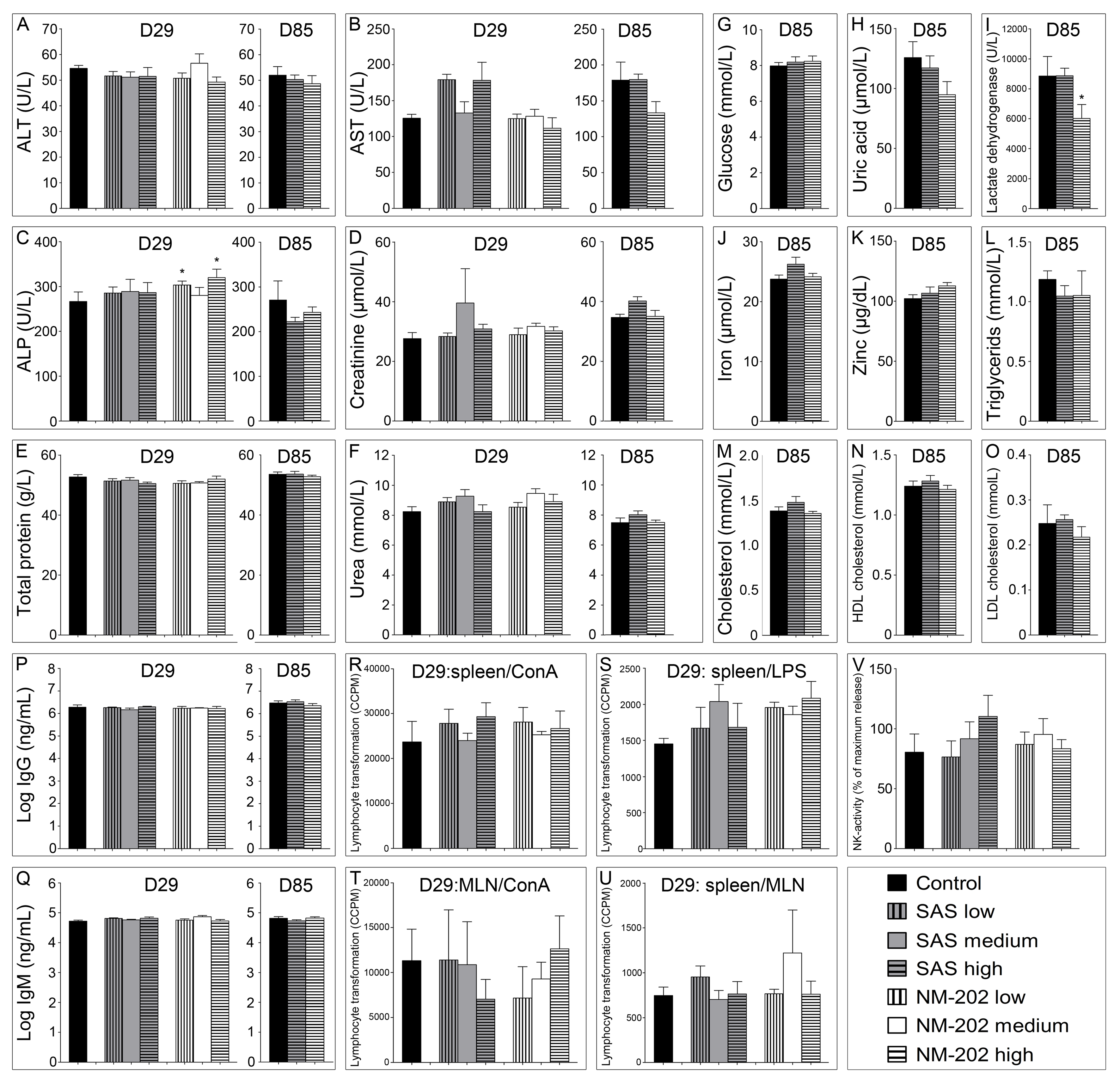
**

**Figure S2:** Systemic and immunotoxic responses in SAS and NM-202 treated animals. (A-O) Concentrations of biochemical indicators in serum after 28- or 84 days of exposure (mean ± SEM; n=5). (P, Q) Antibody levels in serum (mean ± SEM; n=5) after 28- or 84 days of exposure. (R, U) Proliferation of lymphocytes (mean ± SEM; n=5), isolated from the spleen and mesenteric lymph nodes (MLN) after 28 days of exposure. Results are expressed in counts per min. originating from the incorporation of radioactively labelled thymidine. (V) Activity of NK-cells (mean ± SEM; n=5), isolated from the spleen. Results are expressed as a percentage of the maximal release of radioactivity from 51Cr-labelled YAC-1 target cells. Taken together, these results indicate that there is no apparent systemic or immunotoxic effect due to the treatment. Abbreviations: ALP: alkaline phosphatase, ALT: alanine transaminase, AST: aspartate transaminase, HDL/LDL high- and low-density lipoprotein, SEM: standard error of the mean. * Significant difference *versus* the control at the same day (p<0.05).

**
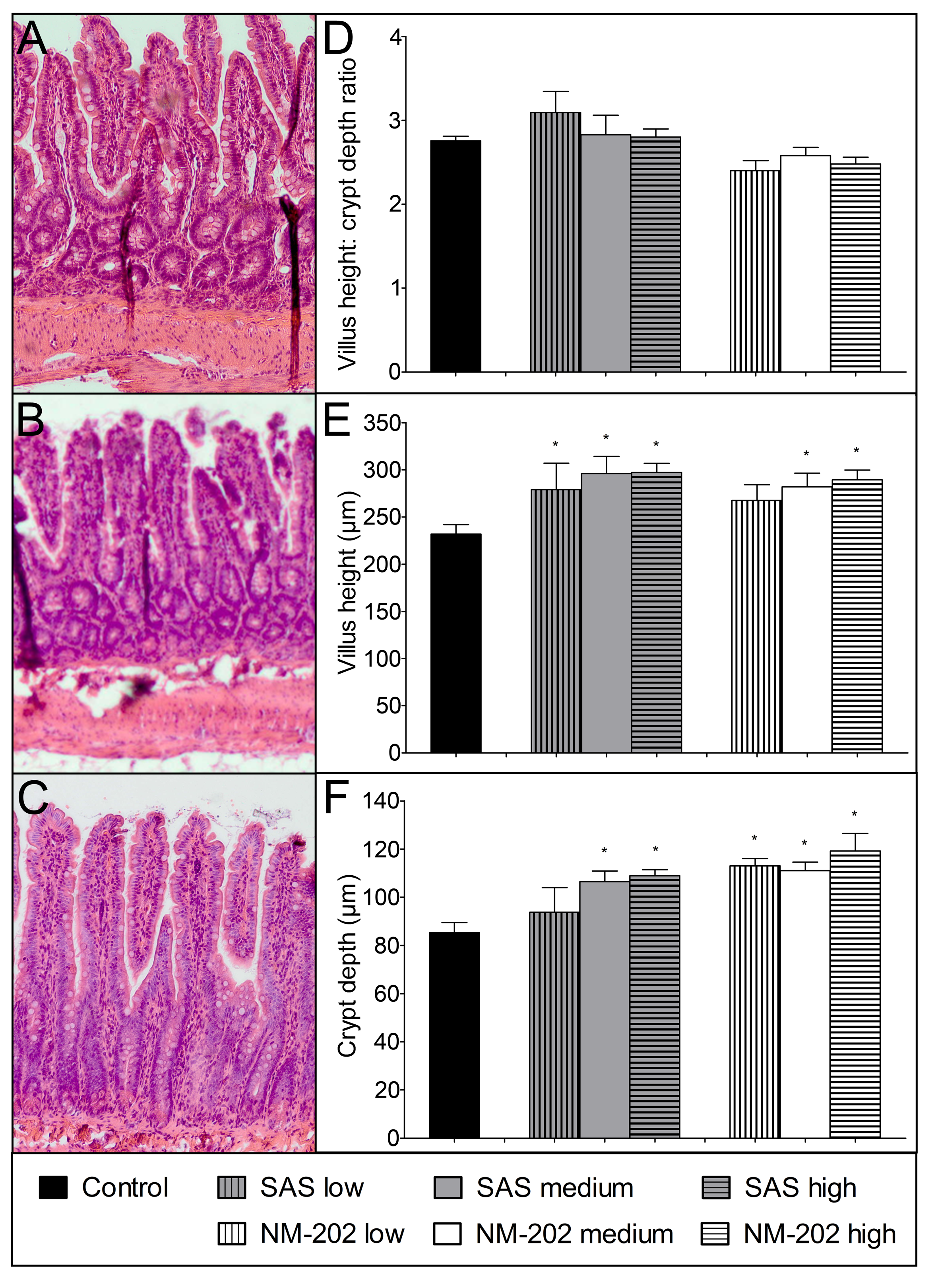
**

**Figure S3**: Quantitative histopathological evaluation of jejunum from animals treated with SAS or NM-202 for 28 days. Cross-sections of jejunum (H&E stained) of (A) control, (B) SAS low, and (C) NM-202 low exposed animals, at 2.5x magnification, showing villi and crypts. Effect of the different treatments (mean ± SEM; n=5) on (D) villus height: crypt depth ratio, (E) villus height, and (F) crypt depth. The villus height is significantly increased in all groups *versus* the control, except for the NM-202 low group. The crypt depths are significantly increased in all groups except for the SAS low group. Since both the villus height and the crypt depth increased in almost all groups there were no significantly differences in the ratio between the villus height and crypth depth. An increased villus:crypt ratio, in combination with an increased villus height suggests a well differentiated, active tissue. Abbreviations: SEM: standard error of the mean. ***** Significant difference *vs.* the control (p<0.05).

**Methods**

**XPS characterization**

Briefly, compressed dry pellets of sample were mounted on the sample holder using a double sided ultra-high vacuum compatible (Si free) copper adhesive tape. Clean Al foil was used to press the material onto the tape forming a uniform and continuous film. All sample handling was done using nitrile powder free gloves. XPS measurements were performed using an AXIS ULTRA Spectrometer (KRATOS Analytical, UK), which was calibrated using a clean, pure Au/Cu sample and pure Ag sample (99.99%). Measured values for electron binding energies (BE) were 84.00+/- 0.02 eV, and 932.00+/-0.05eV [1]. Samples were irradiated with monochromatic AlK α X-rays (hν=1486.6eV) using an X-ray spot size of 400x700 µm^2^ and a take-off angle of 90° with respect to the sample surface. The base- and operating pressure of the instrument were better than 1x10^-8^ Torr and 3x10^-8^ Torr respectively. To compensate for surface charging, a filament (I=1.9A) was used and all spectra were corrected by setting hydrocarbon to 285.00eV. A survey spectrum (0-1110eV) was recorded at pass energy of 160eV for each sample from which the surface chemical compositions (at%) were determined. Also, one set of high-resolution spectra (PE=20eV) was recorded for each sample. Data processing was performed using Vision2 software (Kratos Analytical, UK). Sample compositions were obtained from the survey spectra after linear background subtraction, using the Relative Sensitivity Factors included in the software derived from Scofield cross-sections. Curve fitting of core level peaks was carried out using the same initial parameters and inter-peak constrains to reduce scattering. The core level envelopes were fitted with Gaussian- Lorentian function (G/L=30) and variable full width half maximum.

1. Briggs D. SMP: **Practical surface analysis by Auger and x- ray photoelectron spectroscopy.** New York: Wiley 1983, 1-533.
